# Evolution of prudent predation in complex food webs

**DOI:** 10.1111/ele.13979

**Published:** 2022-03-01

**Authors:** Orestes U. Gutiérrez Al‐Khudhairy, Axel G. Rossberg

**Affiliations:** ^1^ 4617 School of Biological and Behavioural Sciences Queen Mary University of London London UK

**Keywords:** allometric scaling, analytic theory, apparent competition, biogeography, ecological modelling, evolution of cooperation, functional responses, invasive alien species, spawner–recruitment relations, trophic interaction strength

## Abstract

Prudent predators catch sufficient prey to sustain their populations but not as much as to undermine their populations’ survival. The idea that predators evolve to be prudent has been dismissed in the 1970s, but the arguments invoked then are untenable in the light of modern evolution theory. The evolution of prudent predation has repeatedly been demonstrated in two‐species predator–prey metacommunity models. However, the vigorous population fluctuations that these models predict are not widely observed. Here we show that in complex model food webs prudent predation evolves as a result of consumer‐mediated (‘apparent’) competitive exclusion of resources, which disadvantages aggressive consumers and does not generate such fluctuations. We make testable predictions for empirical signatures of this mechanism and its outcomes. Then we discuss how these predictions are borne out across freshwater, marine and terrestrial ecosystems. Demonstrating explanatory power of evolved prudent predation well beyond the question of predator–prey coexistence, the predicted signatures explain unexpected declines of invasive alien species, the shape of stock–recruitment relations of fish, and the clearance rates of pelagic consumers across the latitudinal gradient and 15 orders of magnitude in body mass. Specific research to further test this theory is proposed.

## Graphical abstract

The authors propose a new theory explaining how predators can evolve to catch sufficient prey to sustain their populations but not as much as to undermine their populations’ survival. The key mechanism is consumer‐mediated ("apparent'") competitive exclusion of resources, which disadvantages aggressive predators in the metacommunity context by leaving them vulnerable to sudden extirpation in local communities. Testable empirical signatures of this mechanism and its outcomes include known but hitherto unexplained phenomena in fisheries science, invasion ecology, and the study of allometric scaling laws.

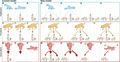

## INTRODUCTION

**FIGURE 1:**
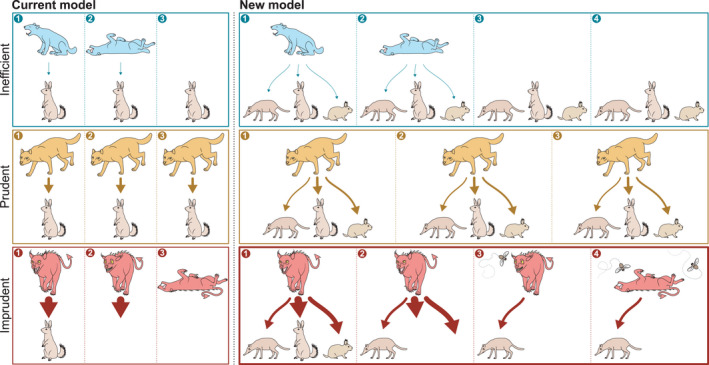
Comparison of current and new model of selection for prudent predation. The six panels illustrate (numbered) sequences of events. Arrows point from consumers to resources, arrow width indicates attack rate. According to both models, consumers that are either too inefficient (top row) or imprudently aggressive (bottom row)—approaching Darwinian Daemons—easily get extirpated. Prudent consumers (middle row) persist for longer. Contrasting the prevailing model, our new model predicts that imprudent consumers feeding on multiple resources ‘hang on’ after extirpating their most important resources, feeding on less suitable resources that persist. This, however, leaves them in a weak position. Any subsequent change in community structure, for example spread of a disease (symbolised by flies), can push them over the edge, leading to extirpation. The resulting separation of the ultimate and the proximate cause of extirpation, seen similarly for invasive alien consumers, is a signature of the new model. Illustration: Rebecca Gelernter/Near Bird Studios

Altruism, the display of traits detrimental to the fitness of individuals but beneficial to others (Boorman & Levitt, 1980), is observed throughout the living world, including plants (Dudley, 2015), non‐human mammals (Schino & Aureli, 2010) and bacteria (Refardt et al., 2013). Controversial, however, remains whether it also occurs in the most relentless kind of ecological interaction, foraging on living resources. We shall call consumers (species feeding on living resources, e.g. predators, herbivores) *prudent* (Slobodkin, 1960) if they feed at a rate sufficient to sustain their populations but not so much that resource overexploitation would become detrimental to their populations’ persistence (Figure 1). We will speak of *evolved prudence* (or similar) when prudence arises through the consumer’s adaptation to its native resource community by mutation and selection.

The idea that consumers have evolved to be prudent was proposed by Slobodkin (1960) based on his observation that transfer efficiencies observed by Lindeman (1942) and others in the field were close to the highest efficiency artificial harvesting of grazers can achieve in the laboratory. Slobodkin’s hypothesis was, however, criticised by Maynard Smith and Slatkin (1973) because, in their view, it led to “the necessity for supposing the evolution of prudence by group selection” and group selection would be unlikely to occur. Attesting to the lasting impact of this view, a similar critique of Slobodkin’s hypothesis can be found in the *Encyclopedia of Ecology* over 30 years later (Matsuda, 2008).

Meanwhile, however, the evolution of prudent predation has been demonstrated for a variety of two‐species consumer–resource metacommunity models (Figure 1, left; Gilpin, 1975; Haraguchi & Sasaki, 2000; Pels et al., 2002; Rauch et al., 2003; Messinger & Ostling, 2013), including individual‐based models (Goodnight et al., 2008; Mitteldorf et al., 2002; Rand et al., 1995). All these studies demonstrate the emergence of a steady state in which, resulting from the evolutionary adaptation of the consumer’s attack rate (or similar), consumers and resources coexist. These results cast doubt on the theoretical intuition that prudent predation can hardly evolve simply because it requires group selection.

Moreover, it has become clear that the very distinction between ‘kin selection’ (conceptualised as acting on individuals and their relatives; with broader acceptance in the scientific community) and ‘group’ or multilevel selection is not fundamentally one between processes but one between mathematical methods (Lion et al., 2011). For problems where both methods are applicable, they yield the exact same result (Jansen, 2011). A categorical dismissal of evolved prudent predation is therefore now more difficult.

There is another, more profound issue, which has puzzled ecologists since Nicholson (1933) and Gause (1934): consumer and resource coexistence in simple model systems, both experimental and mathematical, requires careful adjustment of parameters. In the classical consumer–resource model of Rosenzweig and MacArthur (1963), for example, the range of the attack rate parameter a where consumer and resource can coexist without population oscillations spans roughly an order of magnitude. Specifically, consumer–resource oscillations occur for values of a that are only $\left(1+\tau^{‐1}\right)$‐times larger than the minimum value required for the consumer to survive, where τ denotes the proportion of time consumers spend at population‐dynamical equilibrium ‘handling’ resources rather than ‘searching for’ resources in the behavioural model underlying the Type II functional response (Holling, 1959). Beyond this range, population minima reached by the resource during oscillations decline exponentially with a, soon leading to extinction of any finite resource population—and subsequent extinction of the consumer. In general, the relevant dimensionless parameter is the product of assimilation efficiency, attack rate and resource carrying capacity (all in biomass units) divided by the consumer’s rate of biomass loss due to respiration and mortality (below *respiration* + *mortality* rate). To permit coexistence, it must lie between one and some tight, model‐dependent upper limit. To assume that in nature this condition is regularly satisfied by pure chance would be implausible. Considering the large variety of known consumer strategies to locate, chase, trap and/or subdue resources, of resource strategies to hide, escape and defend themselves, and of typical resource abundances, variation in the parameter over much more than two orders of magnitude would be expected. How then can consumers and resources coexist in the wild?

This problem has been intensely discussed among theorists during the 1970s, but without satisfactory resolution (Slatkin & Maynard Smith, 1979). Next to invocation of some form of altruism, two other important lines of thought were developed: (i) joint evolutionary dynamics of consumer and resource and (ii) structured population models. Exploring the first, Schaffer and Rosenzweig (1978) asked under which conditions the joint evolution of consumer and resource in the Rosenzweig‐MacArthur model leads to an evolutionary stable steady state with consumer–resource coexistence. They found this to be possible when, measured on the relevant scale, the resource evolves faster than the consumer. An argument for why consumers should evolve slower than their resource is the ‘Life‐Dinner Principle’ (Dawkins & Krebs, 1979): for the resource, it is about survival, for the consumer just a meal. The empirical evidence, however, is to the contrary: studies of food‐web topologies, in conjunction with phylogenetic data (Bersier & Kehrli, 2008; Eklöf & Stouffer, 2016) or on their own (Rossberg et al., 2006), consistently show that in a joint niche space in which consumer traits need to match resource traits to yield maximum attack rate (Rossberg et al., 2010), resources tend to evolve much slower than consumers.

The second line of thought considers structured population models. Stage‐structured population models (Maynard Smith & Slatkin, 1973; Slobodkin, 1974) can mitigate the problem of overexploitation, but do not appear to ultimately resolve it (Maiorana, 1976; Maynard Smith & Slatkin, 1973; Mertz & Wade, 1976; Slatkin & Maynard Smith, 1979). More promising are spatially structured models (Hastings, 1977; Hilborn, 1975). In agreement with early intuition (Nicholson, 1933; Nicholson & Bailey, 1935) and experiments pioneered by Huffaker (1958) and Pimentel et al. (1963), repeated recolonisation can permit metapopulations of consumers and resources to coexist even when consumers locally extirpate their resources. However, while this mechanism relaxes constraints on parameters for coexistence, it does not entirely eliminate them. Consumers still go extinct if their attack rates are too high or too low for a given dispersal rate (Mitteldorf et al., 2002) or their dispersal rates too high or too low for a given attack rate (Hilborn, 1975). Furthermore, if one permits the consumer’s attack rate to evolve in such models—and why not—it naturally adjusts itself at values permitting coexistence (Gilpin, 1975; Mitteldorf et al., 2002); that is, prudence evolves. Similar trends are observed in experiments (Pimentel et al., 1963). Such metacommunity models, therefore, hardly serve as alternatives to evolved prudence in explaining consumer–resource coexistence.

One problem, however, is shared by these metacommunity models of one consumer and one resource species, that is, with *monophagous* consumers—with or without the evolution of attack rate. The scenarios of local boom‐bust cycles they predict might describe pests raging across landscapes but are not sufficiently common to support them as general explanations for consumer–resource coexistence in nature (Maynard Smith & Slatkin, 1973; Taylor, 1990). Empirical reports of such boom‐bust cycles for closely associated consumer–resource pairs present them as ecological curiosities rather than a generic phenomenon (Dempster, 1971; Eber & Brandl, 1994; Schöps, 2002; Johst & Schöps, 2003, further examples reviewed by Taylor, 1991). Hence, there remain grounds for scepticism.

Persistent scepticism about evolved prudence is not only evident from direct criticism (Matsuda, 2008) and recent proposals that attack rates follow from fundamental physical and physiological constraints (Hirt et al., 2020; Ho et al., 2019; Pawar et al., 2012; Portalier et al., 2019). It is also implied in assumptions of physiological trade‐offs or pleiotropy in evolutionary models of consumer–resource interactions (Fleischer et al., 2018; Schreiber et al., 2018; van Velzen & Gaedke, 2017). Simple consumer–resource models with evolving attack rates often require such trade‐offs to avoid ever increasing attack rates and the resulting resource extirpation (Abrams, 2000; Gibert & Yeakel, 2019). The trade‐offs usually limit the range over which attack rate can vary in these models, implying that physiological constraints prevent attack rates from becoming too large for coexistence, rather than ecological constraints intrinsic to consumer–resource interaction.

A clue for resolving the mismatch in phenomenology described above comes from studies of the closely related ‘paradox of enrichment’ (Rosenzweig, 1971). We note that, in a review of this subject by Roy and Chattopadhyay (2007), all cited experiments where enrichment (i.e. increased resource carrying capacity) led to stronger oscillations used only one resource species, while all those where this was not observed involved multiple resources (excluding Kirk (1998), who artificially stabilised resource abundance). With multiple resources, enrichment, instead of inducing oscillations, led to decline of the most suitable resources and presumably their replacement by less suitable ones in consumer diets (Persson et al., 2001).

Here we show that a similar process can lead to selection for prudence (Figure 1, bottom right) and overcome the mismatch between the predicted and observed phenomenology of its evolution. We take into account that most consumers are *polyphagous*, feeding on multiple resource, and parts of complex ecological communities continuously turning over in species composition (Dornelas et al., 2014; O’Sullivan et al., 2021a; Yoccoz et al., 2018). The resulting selection mechanism (Figure 1, right) is independent of predator‐prey oscillations (Rosenzweig, 1971) and aligns better with observations.

Early studies demonstrating the evolution of prudence in food webs employed a model (the PDMM, Rossberg et al., 2008; Rossberg, 2013, Sec 22.3) that characterises species by body size and other evolving traits, which in turn jointly determine interactions and interaction strengths. Communities assembled by the PDMM share key properties with marine food webs (Fung et al., 2015). Feeding follows Type II functional responses with prey switching (van Leeuwen et al., 2013; Morozov & Petrovskii, 2013), which can lead to both stable population‐dynamical equilibria (Fung et al., 2015) and complex oscillatory dynamics (Rossberg et al., 2008). As in two‐species models of evolved prudence, attack rates evolve in the PDMM towards a steady state where the dimensionless quantity defined above is close to one (Rossberg et al., 2008). The insight that prudence can evolve in such a detailed, realistic model is important—but many of these details are inessential. In simple food‐web models with two trophic levels and linear functional responses, studied since MacArthur (1969), the same phenomenon is observed (Rossberg, 2013, Sec. 20.4).

In this class of evolutionary food‐web assembly models, the focal, modelled ecological community represents just one of many patches of a larger metacommunity (Rossberg, 2008, 2013, Secs 9.2–9.3; Rossberg et al., 2008; Powell & McKane, 2009). Species can invade the focal patch from this metacommunity and disperse to other patches until their extirpation through community turnover. These models therefore track only short sections of the fate of any evolving lineage. Yet, making the assumption that the focal patch is statistically representative of the metacommunity as a whole, one can draw conclusions from these models about drivers and outcomes of evolutionary processes.

Using such a model, we will here address three questions: By what mechanism does prudence evolve in food‐web assembly models? What kind of observations would provide evidence that this mechanism is active in nature? To what extent has this evidence been observed?

## THE TWO‐LEVEL LOTKA–VOLTERRA FOOD‐WEB ASSEMBLY MODEL AND ITS DECONSTRUCTION

### The full model

Our working model is a Lotka–Volterra‐type model in which $S_{C}$ consumers forage on a community of $S_{R}$ living resources (Rossberg, 2013, Ch. 20):

(1a) $$\begin{array}{c} \frac{d{\hat{B}}_{j}^{R}}{dt}=\left[r\;\left(1‐\frac{{\hat{B}}_{j}^{R}}{K}\right)‐\sum_{k=1}^{S_{C}}a_{\mathrm{jk}}{\hat{B}}_{k}^{C}\right]{\hat{B}}_{j}^{R\;\;}\;\;\;\;\;\;\;\;\;\;(1\leq j\leq S_{R}), \end{array}$$

(1b) $$\frac{d{\hat{B}}_{k}^{C}}{dt}=\left[\varepsilon \sum_{j=1}^{S_{R}}a_{\mathrm{jk}}{\hat{B}}_{j}^{R}‐\rho_{k}\right]{\hat{B}}_{k}^{C}\;\;\;\;\;\;\;\;\;\;\;\;\;\;\;\;\;\;\;\;\;\;\;\;\left(1\leq k\leq S_{C}\right).$$

Here t is time, ${\hat{B}}_{j}^{R}$ is the time‐dependent population biomass (or biomass density) of the j‐th resource in physical units and ${\hat{B}}_{k}^{C}$ that of the k‐th consumer. For simplicity, we assume identical intrinsic growth rates r and carrying capacities K of resources, absence of direct competition between producers, and identical assimilation efficiencies ε for all consumers. The coefficient $a_{\mathrm{jk}}\geq 0$ represents the attack rate of consumer k on resource j. Finally, $\rho_{k}$ denotes the respiration+mortality rate (dimension 1/Time) of consumer k. In most cases, we assume identical $\rho_{k}=\rho$ for all consumers.

To simplify analytic calculations, we express attack rates by the dimensionless coefficients $H_{\mathrm{jk}}=\alpha_{0k}a_{\mathrm{jk}}$, with $\alpha_{0k}=\varepsilon K/\rho_{k}$ (abbreviated to $\alpha_{0}$ if all $\rho_{k}=\rho$), and measure resource biomass $B_{j}^{R}$ in units of K and consumer biomass $B_{k}^{C}$ in units of $\alpha_{0k}r$ (Table 1), yielding the equivalent system

(2a) $$\begin{array}{c} \frac{dB_{j}^{R}}{dt}=r\left[1‐B_{j}^{R}‐\sum_{k=1}^{S_{C}}H_{\mathrm{jk}}B_{k}^{C}\right]B_{j}^{R}\;\;\;\;\;\;\;\;\left(1\leq j\leq S_{R}\right), \end{array}$$

(2b) $$\begin{array}{c} \frac{dB_{k}^{C}}{dt}=\rho_{k}\left[\sum_{j=1}^{S_{R}}H_{\mathrm{jk}}B_{j}^{R}‐1\right]B_{k}^{C}\;\;\;\;\;\;\;\;\;\;\;\;\;\;\;\;\left(1\leq k\leq S_{C}\right). \end{array}$$

**TABLE 1:** List of symbols and model parameters

Symbol	Description	Value
A	Resident’s growth rate term	$R_{l}‐1$
a, $a_{k}$	Base attack rate (of consumer k)	Equation (3)
$a_{\mathrm{jk}}$	Rate of attack rate of resource j by consumer k	$a_{k}e^{\sigma \xi_{\mathrm{jk}}}$
$\alpha_{0k}$	Attack rate scaling factor	$K\varepsilon \rho_{k}^{‐1}$
B	Resident l’s intraspecific competition term	$\sum_{j}H_{\mathrm{jl}}^{2}$
${\hat{B}}_{j}^{R}$	Resource biomass (density) in physical units	$KB_{j}^{R}$
${\hat{B}}_{k}^{C}$	Consumer biomass (density) in physical units	$\alpha_{0k}rB_{k}^{C}$
$B_{j}^{R}$	Dimensionless resource biomass	
$B_{k}^{C}$	Dimensionless consumer biomass	
b(a)	Rate of successful dispersal to other patches	
β	Abundance scaling factor in ‘birth’ probability formula	0.45
C	Focal species’ growth rate term	$R_{k}‐1$
$C_{m}$	Maintenance food concentration	$\rho_{k}/\left(\varepsilon a_{\mathrm{jk}}\right)$
D	Interspecific competition term	$\sum_{j}H_{\mathrm{jk}}H_{\mathrm{jl}}$
ε	Assimilation efficiency	0.1
$\gamma_{0}$	Mutation bias of base attack rate	$0.8^{1/2}$
$\gamma_{1}$	Mutational variation of base attack rate	$1.3^{1/2}$
$H_{\mathrm{jk}}$	Dimensionless attack rate of resource j by consumer k	$\alpha_{0k}a_{\mathrm{jk}}$
K	Resource carrying capacity in absence of consumers	1
L(a)	Mean time to extirpation from a community	
$M_{\min}$	Extirpation threshold (dimensionless biomass)	$10^{‐5}$
$P_{\mathrm{inv}}\left(a\right)$	Invasion probability	
$\rho =\rho_{k}$	Rate of consumer biomass loss by respiration and mortality	0.1
R(a)	Mean number of successful dispersals to other patches	L(a)b(a)
$R_{k}$	Basic reproduction number of *individuals* of species k	$\sum_{j}H_{\mathrm{jk}}$
r	Resource intrinsic per capita growth rate	1
Rec	Fish population recruitment rate	
$S_{C}$	Consumer species richness	
$S_{R}$	Resource species richness	
SSB	Fish population standing stock biomass	
σ	Standard deviation of log attack rates	4
t	Time	
$\zeta ,\xi_{\mathrm{jk}}$	Standard normal random variates	N(0,1)

After initialising model communities with $S_{R}=20$ resources and $S_{C}=10$ consumers, assembly proceeds through iterative invasion of random species (Caldarelli et al., 1998; Post & Pimm, 1983). At the start of each iteration, it is first decided at random, with equal probability, whether the newly invading species will be a consumer or a resource. Candidate species of the chosen type are then sampled at random as described below until one is found that can invade the community (i.e. for which the term in brackets in Equation (2) is positive). After adding this species to the community with an initial biomass of $M_{\min}$, population dynamics are simulated until a new equilibrium is reached (based on the criteria given by Fung et al., 2013) with a cut‐off at $10^{5}$ unit times, while species whose populations fall below $M_{\min}$ are removed as extirpated. Then a new iteration is started.

Note that, for hypothetical resources j in equilibrium that are not fed upon, $B_{j}^{R}=1$. Hence, by Equation (2b), a consumer k would have population growth rate $\rho_{k}\left[\sum_{j=1}^{S_{R}}H_{\mathrm{jk}}‐1\right]$ if it would not share resources with other consumers and its own abundance $B_{k}^{C}$ was too low to affect its resource populations. This becomes $\sum_{j=1}^{S_{R}}H_{\mathrm{jk}}‐1$ when measuring growth rate in units of $\rho_{k}$. In this expression, the sum $\sum_{j=1}^{S_{R}}H_{\mathrm{jk}}=\varepsilon K\rho_{k}^{‐1}\sum_{j=1}^{S_{R}}a_{\mathrm{jk}}$ corresponds to the dimensionless quantity identified in Introduction as being constrained by prudence through the monophagous mechanism (with $S_{R}=1$). We will show that for the new, polyphagous mechanism it plays a similar role. Indeed, defining $R_{k}=\sum_{j=1}^{S_{R}}H_{\mathrm{jk}}$, the classical invasibility criterion (Grainger 2019) implies that $R_{k}>1$ is necessary for consumer–resource coexistence, and below we show that much larger $R_{k}$ are detrimental.

Traditional community models have often been formulated in terms of numerical population sizes rather than population biomasses. We would recover such a formulation here, for example by assuming that all individuals of species k have the same body mass $m_{k}$ and disregarding the contribution of respiration to $\rho_{k}$. Then $R_{k}$ becomes the basic reproduction number (often denoted ‘$R_{0}$’) of consumer k: the mean lifetime number of offspring at low consumer abundance and in absence of interspecific competition (reviewed by Lion & Metz, 2018). Since in the general case $R_{k}$ plays an analogous role, we call $R_{k}$ the *basic reproduction number* here.

### Sampling of new species

To sample candidate invaders into the modelled food web, we recall that it describes one statistically representative patch of many patches forming a metacommunity. In addition, we assume that, as observed in data and models (O’Sullivan et al., 2021b), the proportion of patches occupied by most species is small. The main direct and indirect interaction partners of species invading the focal patch will therefore generally be different from those in previous source patches, so previous adaptations of consumers to specific resource species, and vice versa, play no essential role. This allows us to sample the interaction strengths $a_{\mathrm{jk}}$ between invading consumers and resident resources, and vice versa, at random. We only account for inheritance of the overall magnitude of the attack rates of invading consumers, as this, we shall see, affects the long‐term fate of their lineages.

Each consumer k is therefore assigned a so‐called *base attack rate* trait $a_{k}$, inherited from the ancestral source population, which controls the magnitude of the pairwise attack rates $a_{\mathrm{jk}}$. Technically, the base attack rate $a_{k}$ enters as a scaling factor in the expression for pairwise attack rates, Equation (4) below (see detailed motivation in Appendix S1 in Supporting Information). Assuming that the distribution of base attack rates in the focal community is representative of the distribution in the metacommunity as a whole, we sample the base attack rate of the ancestral source population of a new invader from the distribution within the focal community. Permitting in addition random mutations of the base attack rate to occur between the source population and the population established by the new invader, the base attack rates of invading consumers species k is sampled as follows:

(3) $$a_{k}=\gamma_{0}\gamma_{1}^{\zeta }a_{l}.$$

Here l is the index of one of the $S_{C}$ resident consumers, sampled at random, ζ is a standard normally distributed random number (both sampled anew for each candidate consumer), and the two parameters $\gamma_{0}>0$ and $\gamma_{1}>1$ control bias (sensu Pomiankowski et al., 1991) and size, respectively, of mutations of base attack rate in the model. We choose $\gamma_{0}<1$, to represent degeneration of traits (accumulation of deleterious genetic mutations) in the absence of selection. For our choices of model parameter (Table 1), about 20% of mutations raise base attack rate by Equation (3) ($a_{k}>a_{l}$), which is plenty in view of observed distributions of fitness effects of mutations (Castellano et al., 2019; Eyre‐Walker & Keightley, 2007). The 10 consumers at the start of a model run are all assigned the same initial base attack rate. The attack rates $a_{\mathrm{jk}}$ for newly invading consumers k or resources j are then sampled from log‐normal distributions scaled by $a_{k}$, that is,

(4) $$a_{\mathrm{jk}}=a_{k}e^{\sigma \xi_{\mathrm{jk}}}\;\;\;\;\mathrm{or}\;\;\;\;H_{\mathrm{jk}}=\alpha_{0}a_{\mathrm{jk}}=\alpha_{0}a_{k}e^{\sigma \xi_{\mathrm{jk}}},$$

with independent standard‐normally distributed $\xi_{\mathrm{jk}}$ ($1\leq j\leq S_{R}$); see Appendix S1. The spread σ of the log‐normal distribution is a measure of consumer specialisation (Rossberg et al., 2011) and kept fixed throughout the simulations.

This scheme for sampling the base attack rates of invaders is an adaptation of techniques known as ‘mean‐field’ (or ‘self‐consistent’) approximations, where a single unit is assumed to be statistically representative of a network of interacting units. We checked this approximation in Appendix S6 by confirming that, within numerical errors, the evolutionary stable base attack rate attained by a single species evolving in a corresponding metapopulation model equals the mean base attack rate in the steady state of our food‐web model.

Using Equations (3) and (4), we avoid setting an inherent scale for attack rates. The magnitude of attack rates is controlled by $a_{k}$, and evolution of $a_{k}$ according to Equation (3) is scale free: it is invariant under multiplication of all $a_{k}$, $a_{l}$ by a constant factor. We shall see that the evolutionary stable magnitude of $a_{k}$ is ultimately determined at the ecosystem level.

### The deconstructed model formulation

To gain a better understanding of the processes operating during community assembly and turnover, we developed a novel *deconstructed formulation* of this model. Population dynamics are broken up into a sequence of phases that permit approximate analytic descriptions, thus avoiding simulation of the system of ODEs (2). In Box 1, we named these in analogy to phases of ecological invasions (without claiming identity), as distinguished by Lockwood et al. (2013) and Reise et al. (2006). The analogies become clearest when interpreting our model as describing an island community that is occasionally colonised by species from other islands.

Box 1 **The deconstructed formulation of our community assembly model. Conditions (13)–(17) are derived in Appendix S2**

1. Initialise the model community with a small set of randomly sampled consumers and resources ($S_{C}=10$ and $S_{R}=20$). The subsequent addition of species, resulting in community assembly and turnover, occurs by:
2. **Transport (i)**: Sample, with equal probability, whether the next species to invade is a consumer or a resource.
3. If a consumer is to invade:
  - **Transport (ii)**: Sample the base attack rate and interaction coefficients $H_{\mathrm{jk}}$ for a candidate invader k as described in the section ‘Sampling of new species’.
  - **Establishment**: Test whether this consumer can invade using first the criterion that the consumer should satisfy the **invasibility criterion**
(13) $$\sum_{j}^{S_{R}}H_{\mathrm{jk}}‐1>0$$
    as a minimum requirement for consumer k to persist, and then the (stronger but computationally more expensive) requirement that it should not get extirpated through **exploitative competition** with any of the resident consumers l according to
(14) $$\sum_{j}^{S_{R}}H_{\mathrm{jk}}‐1<\left(\sum_{j}^{S_{R}}H_{\mathrm{jk}}H_{\mathrm{jl}}\right)\frac{\sum_{j}^{S_{R}}H_{\mathrm{jl}}‐1}{\sum_{j}^{S_{R}}H_{\mathrm{jl}}^{2}}.$$
    If consumer k cannot invade, repeat from Step 3a until a consumer is sampled that can.
  - **Spread (within community)**: Remove all of the invading consumer’s resources j that get **overexploited** during consumer k’s early boom phase, which happens when
(15) $$H_{\mathrm{jk}}>‐\log\left(M_{\min}\right).$$
  - **Bust after boom**: If the invading consumer now fails the invasibility criterion, Equation (13), remove it and continue with Step 5.
  - **Impact (resource serial extirpations)**: If
(16) $$\sum_{j}^{S_{R}}H_{\mathrm{jk}}‐1>\frac{\sum_{j}^{S_{R}}H_{\mathrm{jk}}^{2}}{\max_{j}\left(H_{\mathrm{jk}}\right)},$$
    indicating the extirpation of j’s main resource through consumer‐mediated (‘apparent’) competition, remove that resource and repeat Step 3e.
4. If a resource is to invade:
  - **Transport (ii)**: Sample the resource’s interaction coefficients $H_{\mathrm{jk}}$ as described in the section ‘Sampling of new species’ and add it to the community.
  - **Expansion & Impact**: While there are consumers satisfying the condition for consumer mediated resource extirpation, Equation (16), repeat the following:
    - Chose one of these consumers at random and call it l.
    - Remove l’s main resource.
    - Remove any consumers k that now fail to satisfy the invasibility criterion, Equation (13).
5. **Adjustment (exploitative competition)**: Test which consumers k satisfy the condition for exploitative competitive exclusion, Equation (14), by any other consumers l. Then remove all that do.
6. **Adjustment (Pyrrhic competition)**: Test which consumers k satisfy the condition for loss in Pyrrhic competition, Equation (17) below, against any other consumers l. Then remove *the main resource* of each k that does.
7. **Adjustment (starvation)**: Remove all consumers that now fail the invasibility criterion, Equation (13).
8. Repeat from Step 2 for a predetermined number of iterations.
  In Step 6, Phyrric competition between consumers k and l (k≠l) leads to extirpation of k’s main resource i if:
(17) $$\left(\sum_{j}^{S_{R}}H_{\mathrm{jk}}^{2}\right)\left(\sum_{j}^{S_{R}}H_{\mathrm{jl}}^{2}\right)‐{\left(\sum_{j}^{S_{R}}H_{\mathrm{jk}}H_{\mathrm{jl}}\right)}^{2}<\;\left(\sum_{j}^{S_{R}}H_{\mathrm{jk}}‐1\right)\left(H_{\mathrm{ik}}\sum_{j}^{S_{R}}H_{\mathrm{jl}}^{2}‐H_{\mathrm{il}}\sum_{j}^{S_{R}}H_{\mathrm{jk}}H_{\mathrm{jl}}\right)+\left(\sum_{j}^{S_{R}}H_{\mathrm{jl}}‐1\right)\left(H_{\mathrm{il}}\sum_{j}^{S_{R}}H_{\mathrm{jk}}^{2}‐H_{\mathrm{ik}}\sum_{j}^{S_{R}}H_{\mathrm{jk}}H_{\mathrm{jl}}\right)$$
  N.B.: In Appendix S2, we provide a simple algorithmic formulation of this condition.

Contrasting Law and Morton (1996), our deconstructed formulation does not aim to reproduce the dynamics of the full model in all detail, just its system‐level phenomenology. For this, surprisingly coarse approximations are sufficient. These build on the observation that only few resources tend to contribute sizably to a consumer’s diet (Rossberg et al., 2011; Rossberg, 2013, Ch. 12), which we reproduce by our choice of model parameters (Table 1, see Rossberg, 2013, Chs. 11, 12 for detailed discussion). This justifies the simplifying assumption that at most one other consumer needs to be considered to determine a consumer’s persistence with a given set of resources. The full algorithm is described in Box 1. Its formulation highlights the role of the *main resource* of a consumer k, defined as that extant resource for which $H_{\mathrm{jk}}$ is largest over all j.

### Model steady states

**FIGURE 2:**
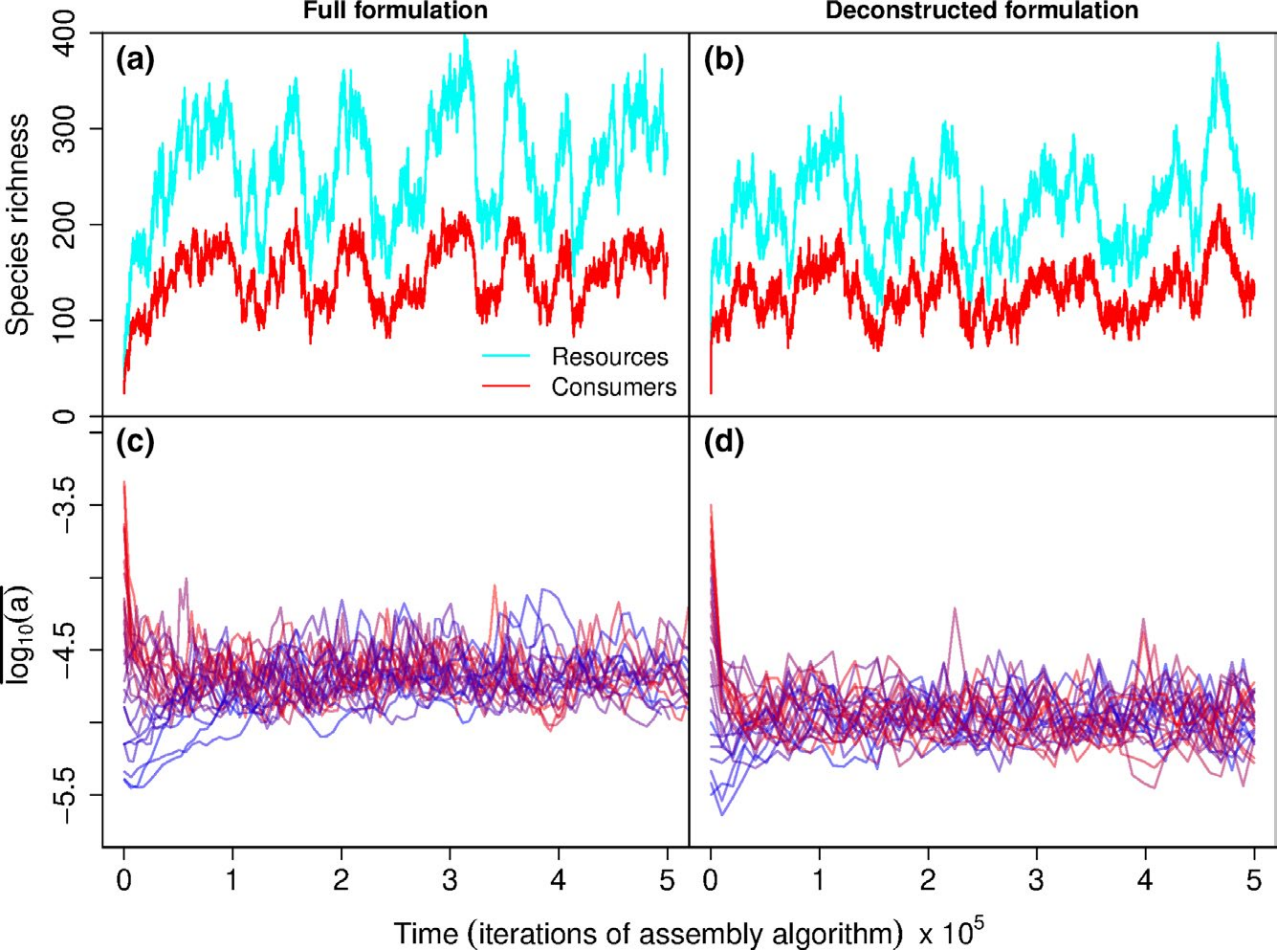
Approaches of full and deconstructed model formulations to steady state. The richness of resources $S_{R}$ (light blue) and consumers $S_{C}$ (red) reaches quasi‐steady states (i.e. they fluctuate around a constant mean) for both full (a) and deconstructed (b) formulation. Furthermore, the quasi‐steady states of both formulations display similar means and patterns of variation. Likewise, community mean base attack rates $\bar{\log_{10}\left(a\right)}$ reaches quasi‐steady states for both (c) full and (d) deconstructed formulation, with steady state values being independent of initial values (colour graduation). With the overline indicating averages, we obtain from the model steady state (between $2\cdot 10^{5}$ and $5\cdot 10^{5}$ iterations), ${\bar{S}}_{R}=260$, ${\bar{S}}_{C}=147$, $\bar{\log_{10}a}=‐4.67$ (full), ${\bar{S}}_{R}=224$, ${\bar{S}}_{C}=125$, $\bar{\log_{10}a}=‐4.96$ (deconstructed)

We compared simulations of full and deconstructed formulations with the same set of parameters (Table 1). As shown in Figure 2a,b, the richness of consumers ($S_{C}$) and resources ($S_{R}$) reached in the steady state is similar for the two formulations, and so is the pattern of richness fluctuations.

In Figure 2c,d, we compare the time series of community mean logarithmic base attack rates $\bar{\log_{10\;}a}$ for both formulations. The evolutionary steady state reached is independent of the $a_{k}$ value of the seeding community of consumers (Figure 2c,d), and differs only slightly between model formulations.

Accounting for the log‐normal distribution of sampled attack rates, Equation (4), steady state means reported in Figure 2 imply that during establishment $R_{k}=\sum_{j}^{S_{R}}H_{\mathrm{jk}}$ is on average $\alpha_{0}a_{k}e^{\sigma^{2}/2}S_{R}\approx 17$ (full) and ≈7 (deconstructed). This is evolved prudence: base attack rates $a_{k}$ always adapt such that basic reproduction numbers $R_{k}$ stabilise at values greater but not much greater than 1, despite variation in initial conditions for $a_{k}$ by a factor 100 in our simulations (and a factor 10,000 in similar simulations by Rossberg, 2013, Ch. 20).

## HOW PRUDENCE EVOLVES IN OUR MODEL

To uncover how prudence evolves in our model, we explore three layers of depth of model analysis. These relate to the evolutionary forces at work, the effect of base attack rate on consumer competitiveness and the restructuring of resource communities by consumers. These analyses are followed by summary and discussion of the full mechanism in a non‐technical language.

### Evolutionary forces

To understand the evolutionary forces leading to prudent predation, we first reconstruct the relevant fitness landscape. Note that while we will speak here of consumer populations as if these were units of selection, the precise formulation would be that the units of selection in our model are consumer individuals invading communities to form new resident populations.

**FIGURE 3:**
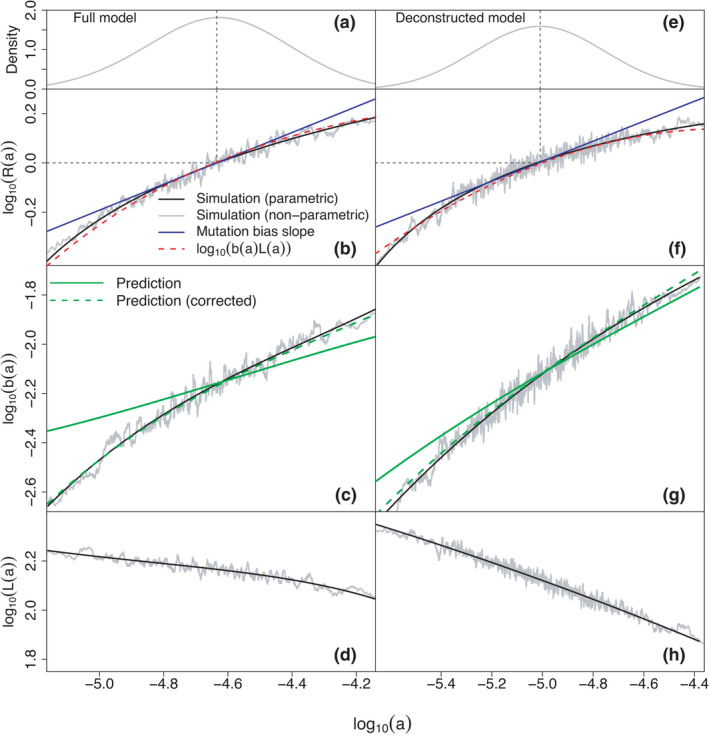
Meta‐community level fitness landscape. Panels (a) and (e) display the distribution of logarithmic base attack rate ($\log_{10}a$) in simulations, with the dashed vertical line representing the simulation mean ($\log_{10}a^{*}$). Panels (b) and (f) represent mean logarithmic population reproductive output ($\log_{10}R\left(a\right)$), which we use as a fitness proxy. The remaining panels display the decomposition of $\log_{10}R\left(a\right)$ into the additive contributions from logarithmic population ‘birth rate’ $\log_{10}b\left(a\right)$ (c), (g) and logarithmic mean population lifetime $\log_{10}L\left(a\right)$ (d), (h) according to Equation (7). We obtained b(a) and L(a) from simulations and verified the decomposition in panels (b) and (f) (red dashed lines). Observe that the curve for $\log_{10}R\left(a\right)$ passes zero and is tangential to the predicted mutation bias slope at $a=a^{*}$, confirming our interpretation of $\log_{10}R\left(a\right)$ as a fitness proxy. Results for the full model [(a)–(e)], Equation (2), are semi‐quantitatively reproduced by the deconstructed formulation [(f)–(h)] (see section ‘The deconstructed model formulation’). All graphs are based on a single simulation with $5\times 10^{5}$ iterations for each model formulation, initiated with base attack rates close to the steady state mean. The first $10^{5}$ iterations were discarded as burn‐ins. Curves in (a) and (e) are obtained using the density function of the R statistical software with standard parameters. The non‐parametric curves in (b), (c), (d), (f), (g) and (h) were computed by taking rolling means of R(a), b(a), L(a) for individual consumers and their $\log_{10}a$ values with a window size equivalent to 1% of the total sample size. The parametric curves are quadratic least‐square fits to the rolling means on double‐logarithmic axes. The predicted ‘birth rates’ in (c) and (g) were calculated according to Equation (S25), the mutation bias slop according to Equation (6)

The approximate normal distribution of logarithmic base attack rates ($\log_{10}\;a$) in the model steady state (Figure 3a,e) suggests an analysis in terms of $\log_{10}a$. We therefore reformulate our model for inheritance of base attack rates, Equation (3), as follows:

(5) $$\log_{10}\;a_{k}=\log_{10}\;\gamma_{0}+\zeta \log_{10\;}\gamma_{1}+\log_{10}\;a_{l}.$$

Hence $\log_{10}\;\gamma_{0}$ represents the size of the mutation bias and ${\left({\text{log}}_{10}\;\gamma_{1}\right)}^{2}$ the mutational variance of $\log_{10}\;a$.

We define R(a) as the average number of other patches populations with base attack rate a successfully colonise, i.e. such a *populations’* ‘mean lifetime reproductive success’. Within the mean‐field approximation, we compute R(a) as the mean number of successful invaders that formally inherit their base attack rates via Equation (3) from a population with base attack rate a.

Unless all species forming a metacommunity have reached an evolutionary steady state, it is unlikely that local community properties attain a steady state over evolutionary time. In Appendix S3, we therefore obtain an evolutionary steady‐state condition from the following steady‐state condition for our community model: denoting by $a^{*}$ the geometric mean of a and by $\mathrm{var}(\log_{10}\;a)$ the variance of $\log_{10}\;a$ in the model steady state, an evolutionary steady state requires that

(6) $${\left.\frac{d\;\log_{10}\;R(a)}{d\;\log_{10}\;a}\right|}_{a=a^{*}}\approx ‐\log_{10}[\exp(1)]\frac{\log_{10}\;\gamma_{0}}{\mathrm{var}(\log_{10}\;a)}.$$

We verify this relation graphically in Figure 3b and f. As expected, for both the full and the deconstructed model (i) the equilibrium condition for species richness, $\log_{10}\;R\left(a^{*}\right)=0$ (i.e. $R(a^{*})=1$), holds, and (ii) the straight line with slope given by Equation (6) is tangential to the graph of $\log_{10}\;R\left(a\right)$ against $\log_{10}\;a$ at $a=a^{*}$. This confirms R(a) as a suitable fitness proxy.

To disentangle the mechanisms determining R(a), define L(a) as the mean time populations with base attack rate a persist in a community, and b(a) as the rate at which they colonise other patches (in the mean‐field approximation: generate new invaders). We can factorise R(a)=b(a)L(a) because the rate at which a population with base attack rate a gives rise to new invasions is in our model independent of the lifetime of this population. Hence

(7) $$\log_{10}\;R\left(a\right)=\log_{10}\;b\left(a\right)+\log_{10}\;L\left(a\right).$$

In Figure 3c,d,g,h (black lines), we show these two components of $\log_{10}\;R\left(a\right)$ for both model formulations, as determined numerically from the model steady states.

The ‘birth rate’ b(a) exhibits an increasing trend with base attack rate a. In fact, the curve can be understood at an analytic level. We included in Figure 3c,g two analytic approximations of b(a). The first is based on a log‐normal approximation for the distribution of the sum $\sum_{j=1}^{S_{R}}H_{\mathrm{jk}}$ in the invasibility criterion, Equation (13). The full calculation, taking into account the mutation bias and the fact that we measure time in units of consumer invasions, is presented in Appendix S4. The resulting dependence of b(a) on $\log_{10}\;a$ has the functional form of a cumulative normal distribution. The second approximation accounts for competition between consumers by multiplying the sum above with a fitting parameter β, which represents the mean scaled biomass of resources encountered by invading consumers. With β=0.25 (full) and β=0.45 (deconstructed), this reproduces the form of b(a) found in our model (Figure 3c,g). This analytic model implies that the graph of $\log_{10}\;b\left(a\right)$ vs. $\log_{10}\;a$ always has a positive slope, is bending downwards, and reaches a plateau for large $\log_{10}\;a$. In the following, we explain why, somewhat counter‐intuitively, the mean population ‘lifetime’ L(a) declines with increasing base attack rate a.

### How base attack rate affects consumer competitiveness

The deconstructed formulation separates different ecological processes with a clarity not offered by simulations of the full model, permitting us to gain insights into the mechanisms controlling population ‘lifetime’ in the model. Reliance on the deconstructed formulation is justified here, because it reproduces the full formulation’s phenomenology well (Figures 2, 3).

Because around 96% of consumer extirpations are triggered either by the competitive exclusion condition, Equation (14), or by failure of the invasibility condition, Equation (13), which implies the former, and because this does not depend much on the base attack rate of the extirpated population (Figure S4), we focus here on the drivers of competitive exclusion.

The deconstructed formulation’s condition for competitive exclusion of a consumer k through exploitative competition with another consumer l, Equation (14), can be rewritten as C<DA/B or

(8) $$\log_{10}C‐\log_{10}D‐\log_{10}A+\log_{10}B<0,$$

with the four named terms

(9) $$A=\sum_{j}^{S_{R}}H_{\mathrm{jl}}‐1,\;\;B=\sum_{j}^{S_{R}}H_{\mathrm{jl}}^{2},\;\;C=\sum_{j}^{S_{R}}H_{\mathrm{jk}}‐1,\;\;D=\sum_{j}^{S_{R}}H_{\mathrm{jk}}H_{\mathrm{jl}}.$$

Terms A and C can be written as $A=R_{l}‐1$ and $C=R_{k}‐1$, respectively, and represent the intrinsic growth rates of the two consumers in units of ρ. Term B quantifies intraspecific competition of l, Term D its competition with k (see also Appendix S2).

**FIGURE 4:**
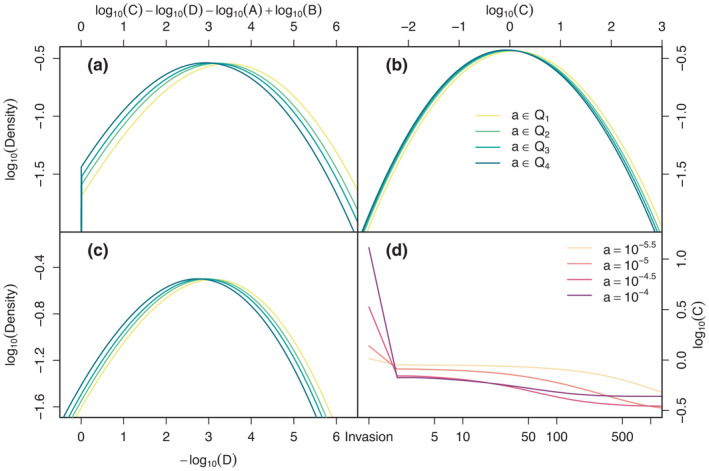
Components of the condition for consumer competitive exclusion in the deconstructed model formulation. Panel (a) represents the distribution of the left‐hand side of condition (8) for competitive exclusion in the steady state of the deconstructed model formulation, panels (b) and (c) two additive contributions defined in Equation (9). Probability densities were computed using the density function of R with bandwidth set to 0.5. They were computed separately conditional to base attack rate a lying in one of the four quartiles of its steady‐state distribution ($Q_{1}$–$Q_{4}$, see legend). Panel (d) shows how the geometric mean ($10^{6}$ replicates) of Terms C changes with each iteration of the serial resource extirpation algorithm of the section ‘Restructuring of resource communities by consumers’, for different base attack rates of the consumer (see Appendix S5 for more detailed results). These results reveal that serial extirpation generates an anomaly in the dependence of Term C (but not D) on a that increases the probability of consumers extirpation with increasing base attack rate a

For random pairs k, l of consumers sampled from the steady state of the deconstructed model formulation, the left‐hand side of Equation (8) follows an approximate truncated normal distribution. In Figure 4a, we show this distribution conditional to base attack rate $a_{k}$ lying within each of the four quartiles of the steady‐state distribution of a (Figure 3e). While the variance does not depend much on $a_{k}$, the mean decreases with increasing $a_{k}$, making competitive exclusion by Equation (8) more likely, consistent with the decreasing trend for mean lifetime in Figure 3h. This trend must be due to the dependencies of Terms C and D on $a_{k}$, because A and B depend only on competitor l.

Figure 4b,c show the corresponding distributions of the additive contributions $\log_{10}\;C$ and $‐\log_{10}\;D$. The mean of $\log_{10}\;C$ *decreases* slightly with increasing $a_{k}$ (linear regression ± S.E.: $\log_{10}\;C=\left(‐0.200\pm 0.002\right)\times \log_{10}\;a_{k}+\mathrm{intercept}$). This is surprising. With attack rates sampled at random following Equation (4), a linear *increase* of $R_{k}$ with $a_{k}$ is expected, implying a slope >1 for the regression. By contrast, the decline of $‐\log_{10}\;D$ with increasing $a_{k}$ ($\log_{10}\;D=\left(0.632\pm 0.006\right)\times \log_{10}\;a+\mathrm{intercept}$) is mostly in line with expectations—for $H_{\mathrm{jk}}$ sampled at random according to Equation (4), $D=\sum_{j}H_{\mathrm{jk}}H_{\mathrm{jl}}$ increases linearly with $a_{k}$.

The key to understanding the surprising decline of mean consumer population lifetime L(a) with increasing a therefore lies in understanding the unexpected absence of an increase of $R_{k}$, and so of Term C, with $a_{k}$, and why this is not reflected in Term D. Both $R_{k}$ and Term D are sums of the attack rates of k over all resources. In Term D, the sum contains what are effectively log‐normally distributed random weighting factors $H_{\mathrm{jl}}$. These can give prominence to resources j in the sum that contribute little to the unweighted sum $R_{k}$, and conversely reduce the weight of the resources dominating $R_{k}$. This suggests a central role of the main resources of k, hinting at consumer‐mediated (or ‘apparent’ *sensu* Holt, 1977) competitive exclusion by Equation (16). We follow this lead.

### Restructuring of resource communities by consumers

To understand how consumer‐mediated competitive exclusion affects $R_{k}$ and C, we devised a further simplification of the deconstructed model. In this model, only one consumer k is considered. Its base attack rate $a_{k}$ is a model parameter, and the number of resource species is fixed. The model, detailed in Box 2, mimics gradual changes through time in a consumer’s resource set in the deconstructed model formulation, but suppresses the possibility of consumer extirpation.

Box 2 **A simplified model of serial resource extirpation**

The model simulates a single consumer k=1 with base attack rate $a_{k}$ in a community of $S_{R}=300$ resources. It is described by the following algorithm:

1. Sample sets of $S_{R}$ scaled attack rates $H_{\mathrm{jk}}$ according to Equation (4) until one is found that satisfies the invasibility criterion, Equation (13). Continue with this set.
2. Record the initial value of $C=\sum_{j}H_{\mathrm{jk}}‐1$ (marked ‘Invasion’ in Figure 4d).
3. Impact: as long as the condition for consumer‐mediated competitive exclusion, Equation (16), is satisfied, remove the resource of j with the largest $H_{\mathrm{jk}}$.
4. Record the value of $C=\sum_{j}H_{\mathrm{jk}}‐1$.
5. Replace the resources removed in Step 3 with new ones, sampling new values $H_{\mathrm{jk}}$ following Equation (4). If no resource was removed in Step 3, chose a random resource i and re‐sample $H_{\mathrm{ik}}$, conditional to satisfaction of the invasibility criterion, Equation (13).
6. Repeat from Step 3 for a predetermined number of iterations.

In Figure 4d, we show averages of sequences of $C=R_{k}‐1$ through time predicted by this algorithm for four different values of $a_{k}$. While at the time of invasion (Step 2 in Box 2) C increases with $a_{k}$ in line with expectation, this order is reversed by the first iteration of consumer‐mediated competitive exclusion (Step 3), which corresponds to the Impact phase of the deconstructed formulation. In subsequent iterations, this reversal is maintained and eventually C becomes largely independent of $a_{k}$ (Figure S2).

In Appendix S5, we present a mathematical analysis of the model state after the first execution of Step 3 (Impact). We take the mathematical limit of large resource richness $S_{R}$, while keeping the expected Gini–Simpson dietary diversity of consumers at the time of invasion fixed at a value 0<ν<1 by adjusting the spread σ of the log‐normal attack‐rate distribution as $\sigma =\nu^{‐1}\sqrt{2\ln S_{R}}$ (Rossberg et al., 2011; Rossberg, 2013, Ch. 11, 12). For large base attack rates $a_{k}$, this leads to

(10) $$C=R_{k}‐1=\sum_{j}^{S_{R}}H_{\mathrm{jk}}‐1=1‐\nu$$

after Impact on average. Convergence of $R_{k}‐1$ to this value with increasing $S_{R}$ is slow and therefore the quantitative prediction by Equation (10) not borne out in practice. But its broader implication holds even for moderate $S_{R}$: after Impact, C will be of the order of magnitude of one even when $a_{k}$ is large (Figure 4d).

With the value of Term C thus constrained, while that of Term D increases with base attack rate on average, the likelihood of competitive exclusion by other consumers increases with a consumer’s base attack rate according to Equation (8). This explains why more aggressive consumers have a shorter mean time to extirpation L (Figure 3h).

### Summary of mechanism

We can now put together the picture of how prudence evolves in our model. Crucial is that during the Impact phase imprudent consumers (those with high base attack rates) extirpate their main resources through consumer‐mediated competitive exclusion. As a result, the basic reproduction number of extant consumers in the model depends only weakly on base attack rate, even though in the initial establishment phase it is proportional to base attack rate. Since the effect of resource extirpation on the strength of competition with other consumers is weaker (competition coefficients increase with increasing base attack rate, Figure 4c), less prudent consumers are more likely to get competitively excluded by other consumers. Imprudence thus causes early extirpation on average.

Characteristic of this process is the separation of the ultimate and proximate causes of extirpation of an imprudent consumer (Figure 1). The *ultimate cause* is extirpation of its resources. However, some other event, the *proximate cause*, is needed to push it over the brink. In our model, this can be invasions of immediate competitors or indirect effects of community turnover, for example through Pyrrhic competition (inspection of simulations shows that both cases occur). In reality, shifts in environmental conditions, arrival of predators or spread of diseases can equally play this role.

Early extirpation of imprudent consumers interacts with other evolutionary forces (Figure 3) as follows: ease of establishment in communities increases with increasing base attack rates, but with diminishing returns. Since high base attack rates are not beneficial for the subsequent long‐term population survival, a moderate mutation bias can thus prevent attack rate evolution beyond values where invasions become likely. As a result, prudence evolves. As demonstrated in Appendix S6, ‘cheaters’, who out‐compete prudent conspecifics as they invade local communities, do not fundamentally undermined this outcome.

The mechanism described above is essentially different from resource overexploitation through simple monophagous consumer–resource interactions. The latter occurs either during the initial Spread phase of invasions (Box 1) or—in models with non‐linear functional responses (Rosenzweig, 1971)—in the course of predator‐prey cycles, and is controlled by some lower cut‐off for viable resource population biomass ($M_{\min}$ in Equation (15)). With our choice of $M_{\min}$, such dynamic resource extirpations followed by extirpation of the consumer are rare (Figure S4). By contrast, the polyphagous mechanism does not depend on such a cut‐off because it operates in population‐dynamical equilibrium.

### The role of the functional response

The core element of this new, polyphagous mechanisms—consumer‐mediated competitive exclusion at high base attack rates—operates in complex food webs despite real‐world complications such as competition amongst producers, omnivory, food‐web loops, and phylogenetic and size structure, as long as the approximation of linear functional responses applies (Rossberg, 2013, Sec. 15.3).

Consumer‐mediated competitive exclusion operates also with Type II functional responses (Grover & Holt, 1998; Křivan & Eisner, 2006) and persists under moderate adaptive foraging (van Leeuwen et al., 2007). Only for the extreme case of optimal foraging (Křivan & Eisner, 2006) it disappears.

Predator‐dependent functional responses, however, which describe a reduction of per‐capita feeding rate with increasing consumer (predator) abundance (Tyutyunov & Titova, 2020), facilitate resource coexistence in situations where consumer‐mediated competitive exclusion would otherwise occur (Coblentz & DeLong, 2020). Predator dependence, which is empirically well documented (Skalski & Gilliam, 2001; DeLong & Vasseur, 2011; Arditi & Ginzburg, 2012; Stouffer & Novak, 2021), thus offers an alternative route to prudence. However, first, this just shifts the problem of explaining consumer–resource coexistence to understanding why and to what extent consumers have evolved to restrain foraging in the presence of competitors. Second, with consumers behaviourally restraining themselves, there is again little fitness benefit in excessively high base attack rates. Prudence in nature could be realised by mixtures of varying composition between adaptation of based attack rates and predator‐dependence of functional responses.

### The analogy with the evolution of virulence

The evolution of prudent predation has an analogy in the evolution of the virulence of infectious diseases (Lion & Boots, 2010), a well‐established phenomenon. According to the classical theory by Anderson and May (1982), evolutionary stable virulence is the outcome of a trade‐off between virulence and transmission rate (Cressler et al., 2016). Virulence, the mortality of infected hosts, corresponds to inverse population ‘lifetime’ 1/L(a) in our model and transmission rate to population ‘birth rate’ b(a). As for infectious diseases, the trade‐off between L(a) and b(a) arising in our model (Figure 3) leads to evolutionary stable values for a, b(a), and L(a).

The major difference to current models of evolutionary epidemiology (Cressler et al., 2016) is the inclusion of mutation bias in Equation (3). For viruses, such bias is well documented (Sanjuán et al., 2004; Silander et al., 2007); its omission in epidemiological models most likely just a nod to parsimony. Indeed, such bias would not fundamentally affect outcomes in most epidemiological model. In our case, this is different. Since b(a) plateaus with increasing a and L(a) declines, R(a)=b(a)L(a) appears to attain a maximum along the a axis, representing an evolutionary stable point even without mutation bias. The corresponding base attack rate a, however, is rather high. It would lead to a decline in resource richness (Rossberg, 2013, Sec. 20.2) and, ultimately, to extirpation of all consumers. Mutation bias is hence a facet of reality our model cannot afford to gloss over.

### Prudence and optimisation

The evolution of prudence leads to basic reproduction numbers $R_{k}$ not much larger than 1 for newly establishing consumers k. At first sight, this appears to contradict decades of research demonstrating that organisms evolve to optimise their metabolism, minimise mortality, maximise their intrinsic population growth rates and so, apparently, maximise $R_{k}$. Here we propose how to resolve this apparent contradiction.

The metapopulation fitness of species is determined not only by their abilities to invade patches and population survival within patches, but also by the rate of dispersal from one patch to others. This rate is controlled not only by dispersal strategy but also by population size within patches. All else equal, larger populations disperse more propagules.

Population biomass in our model is ${\hat{B}}_{k}^{C}=\alpha_{0k}rB_{k}^{C}=\varepsilon Kr\rho_{k}^{‐1}B_{k}^{C}$. In this expression, dimensionless population biomass $B_{k}^{C}$ is independent of respiration+mortality $\rho_{k}$ for given scaled interaction strengths $H_{\mathrm{jl}}$ ($1\leq j\leq S_{R}$, $1\leq l\leq S_{l}$), and K and r are characteristics of the resources. To increase population size, and hence dispersal, consumers can therefore adapt to minimise $\rho_{k}$ while at the same time keeping $R_{k}=\sum_{j}^{S_{R}}H_{\mathrm{jk}}$ in the range consistent with prudence. (A corresponding argument could be made for assimilation efficiency ε.)

**FIGURE 5:**
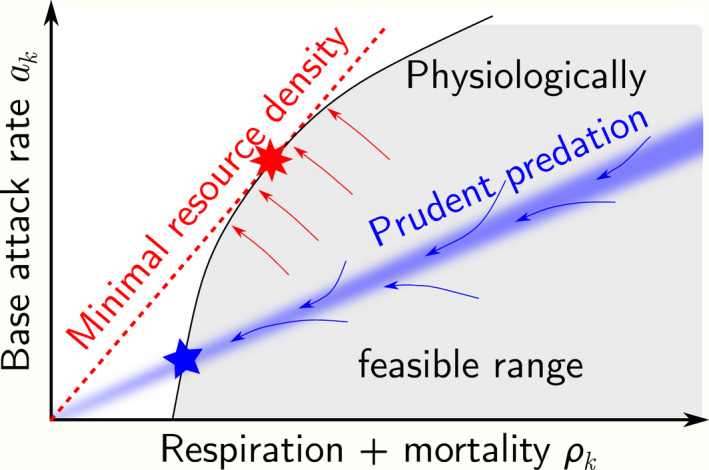
Prudence and optimisation in evolution. The figure schematically illustrates evolutionary forces acting on a single, isolated consumer k (red) feeding on a single resource, and a polyphagous consumer k embedded in a metacommunity (blue). The area shaded in grey indicates the range of physiologically feasible combinations of attack and respiration rates under *ad libitum* feeding. The isolated monophagous consumer will evolve to minimise the abundance of its resource at equilibrium (arrows), controlled by $\rho_{k}/a_{k}$, leading to an evolutionary optimum as indicated by the eight‐pointed star. The polyphagous consumer in a metacommunity will evolve towards prudent predation (range of corresponding $\rho_{k}/a_{k}$ values indicated by blue shading) and also to minimise its respiration+mortality rate (arrows) in order to maximise its abundance. The evolutionary endpoint is then given by the five‐pointed star. Both endpoints are consistent with observations in so far as they represent the limit of physiologically feasible $a_{k}$–$\rho_{k}$ combinations

Figure 5 schematically compares evolutionary forces and the resulting position of the evolutionary stable strategy in the space spanned by $\rho_{k}$ and $a_{k}$ for a prudent consumer (blue) and for a monophagous consumer in an isolated community (red). Both optima are consistent with empirical observations that foraging apparatus and strategies are optimised to maximise base attack rates $a_{k}$ within the limits of given metabolic + mortality costs $\rho_{k}$, and that metabolic and mortality costs are minimised under the constraint of maintaining the biological machinery required to retain a given base attack rate $a_{k}$.

The difference between the two optima lies in the quantitative trade‐off between $\rho_{k}$ and $a_{k}$, that is, the slope of the edge of the physiologically feasible range at the optimum in Figure 5. Empirical work rarely if ever quantifies this trade‐off for comparison with theoretical expectations. Prudent predation therefore cannot be dismissed simply on the grounds that metabolism, longevity and foraging are found to be minimised or maximised in nature with some trade‐off.

## WHAT EMPIRICAL SUPPORT FOR OUR THEORY LOOKS LIKE

We discussed a range of conceivable mechanisms for consumer–resource coexistence. These include resource survival at metapopulation level, resources winning evolutionary arms races, prudence through predator‐dependent functional responses and evolution of prudence via either selection by monophagous boom‐bust cycles or the polyphagous mechanism describe here. A general test for evolved prudence has been proposed by Wilson (1978). To test specifically for the evolution of prudence through the polyphagous mechanism, the theory developed here, we propose to study three kinds of empirical data:

1. Basic reproduction numbers of resident consumers, to test for ecological constraints on this number.
2. Events surrounding invasive alien consumers, to test for separation of ultimate and proximate causes of selection for prudence.
3. Comparisons of minimum required and actual resource densities, to test for manifest prudence.

Below we provide examples of each. Tests 1 and 2 are specific to the polyphagous mechanism. Test 3 excludes metapopulation‐level resource survival and to some extent predator‐dependent functional responses.

### Evidence of ecological constraints on basic reproduction number

**FIGURE 6:**
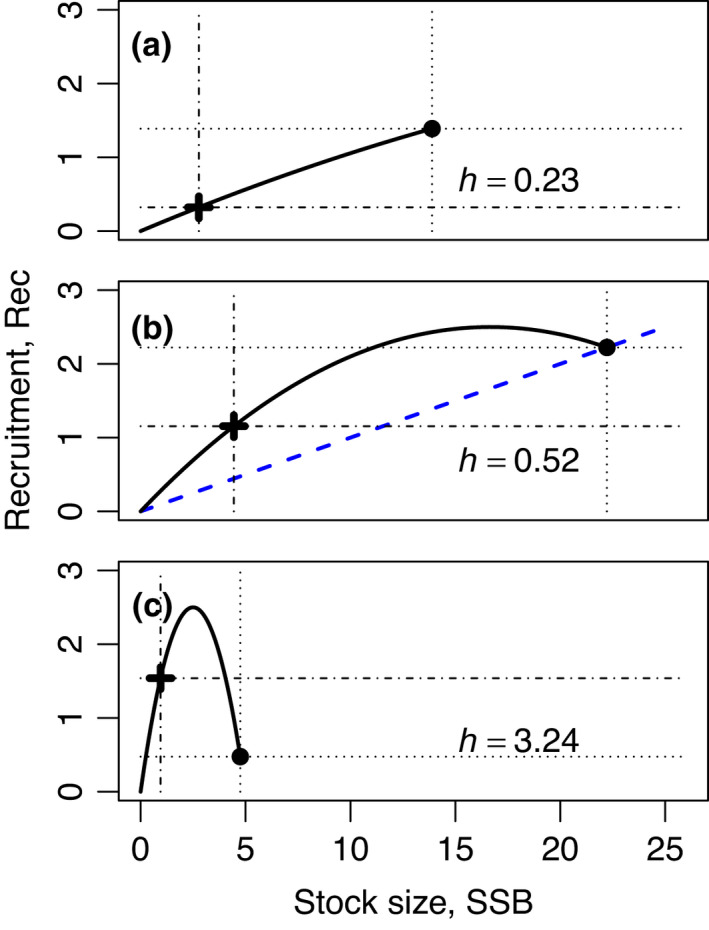
Stock‐recruitment relations and steepness. The figure illustrates the range of possible stock‐recruitment relations (thick lines) that can emerge from a Lotka–Volterra model of a fish stock feeding on a single resource (Appendix S7). Panels a, b and c, correspond to basic reproduction numbers 1.2, 3, and 20, respectively. Specifically, we evaluated the model of Appendix S7 with $a=1.2K^{‐1}$, $3K^{‐1}$, $20K^{‐1}$, the other parameters fixed at s=1, K=100, ε=0.1, ρ=0.1, and fishing mortality F varying from 0 up to the value where the stock goes extinct. Stock size $\mathrm{SSB}={\mathrm{SSB}}_{0}$ and recruitment Rec without fishing (F=0) are indicated by a circle and dotted lines, SSB and Rec at 20% of the unfished stock size by a cross and dash‐dotted lines. The resulting steepness h, defined as the ratio of the two Rec values, is indicated in each panel. To see why steepness and basic reproduction number are closely related, note that for an unfished stock, and hence along the blue dashed line in Panel b, each adult fish has exactly one recruit offspring on average. Basic reproduction number is the factor by which recruitment lies above this line as SSB→0, steepness is 0.2 times this factor at $\mathrm{SSB}=0.2\;\mathrm{SS}B_{0}$. Observed stock‐recruitment relations typically resemble rather Panel b than Panels a or c

A key element of the polyphagous mechanism for evolved prudence is an ecological constraint on the basic reproduction number $R_{k}$ of resident consumers k (after their impact phase) (see section ‘Restructuring of resource communities by consumers’ and Appendix S5). It can be tested by studying what fisheries scientists call *stock*‐*recruitment relations* (Figure 6, thick lines): the functional dependence of the yearly number of newly maturing recruits Rec(SSB) on spawning stock biomass, SSB—the total biomass of a stock’s sexually mature individuals.

With ${\mathrm{SSB}}_{0}$ denoting SSB for the unfished stock, one defines the steepness (Figure 6) of Rec(SSB) as

(11) $$h=\frac{\mathrm{Rec}(0.2\times {\mathrm{SSB}}_{0})}{\mathrm{Rec}({\mathrm{SSB}}_{0})}.$$

Steepness is closely related to basic reproduction number (Figure 6). In Appendix S7, we show that for a single stock k feeding on multiple resources in a Lotka–Volterra model

(12) $$h_{k}=\frac{1}{25}\left(1+4R_{k}\right).$$

The predicted ecological constraint on $R_{k}$ thus implies a constraint on steepness $h_{k}$ (see also Myers et al., 1999).

Is this constraint observed? For fish stocks, yes. Following the realisation that steepness attains preferred values across stocks (McAllister et al., 1994; Punt et al., 1994), priors for steepness are now regularly used to estimate stock‐recruitment relations for data‐poor stocks (Punt & Dorn, 2014). In simple cases, a fixed value for h is used.

This preference for steepness to attain certain values could never be explained (He et al., 2006; Myers et al., 1999). Ginzburg et al. (2010) argued that, for annual or age‐structured populations (Tuljapurkar et al., 1994), periodic or chaotic oscillations can set in at large $R_{k}$, independent of the detailed nature of density dependence, thus potentially selecting against large $R_{k}$. However, for such oscillations to lead to extirpations, and so selection, their amplitude would need to be much larger than anything observed in the fisheries context. Our theory provides a more natural explanation.

Quantitative comparisons of steepness require fixing the functional form of the fitted stock‐recruitment relation (Munyandorero, 2020). Typically, the Beverton–Holt model is used ($\mathrm{Rec}=c_{1}\mathrm{SSB}/\left(1+c_{2}\mathrm{SSB}\right)$ with parameters $c_{1}$, $c_{2}$), for which steepness priors tend to have a mode near h=0.8 (McAllister et al., 1994; Munyandorero, 2020; Shertzer & Conn, 2012; Thorson et al., 2019; Zhou et al., 2012). Remarkably, this mode near h=0.8 was found also in a food‐web model of interacting size‐structured fish populations and their resources (Rossberg et al., 2013), suggesting that, indeed, h=0.8 reflects the ecological constraint on $R_{k}$ we identified.

### Evidence of operation of the polyphagous selection mechanism

By definition, invasive alien species cause harm to the ecosystems they invade, often through predation or grazing. Invasive consumers appear to have base attack rates too high for the invaded ecosystems to sustain. Our theory predicts a series of tell‐tale signatures that should be observable when imprudent alien polyphagous consumers invade local communities:

1. Fast initial population growth, indicative of an imprudent alien consumer.
2. A strong impact on the resource community, involving resource extirpations or resource depletion to low levels sustained by immigration (i.e. mass effects, Shmida & Wilson, 1985). This might go along with exclusion of competing consumers.
3. A halt in population growth, potentially with subsequent decline, after which the invader’s population stabilises.
4. Further local decline or even extirpation of the invader’s population, explained through the (re‐)emergence of competitors, other rather unsuspicious causes, or unexplained.

That is, we are not only predicting “population crashes of established introduced species”, reviewed by Simberloff and Gibbons (2004), but a more detailed pattern that evidences the temporal separation of the ultimate cause (2) from the proximate cause (4) of population collapse. This separation is highly specific to the mechanism we propose. In particular, it does not arise with the monophagous counterpart.

We shall discuss four well‐studied examples of invasive alien consumers where these signatures have been fully or partially documented (Table 2). This serves not only to illustrate how these signatures manifest themselves in the field but also demonstrates that observation of what we predict is not unheard of. A careful meta‐analysis would be required to establish how common documentation of these signatures is and to what extent absence of their documentation is due to incomplete observation or reporting.

**TABLE 2:** Examples of observed signatures of the operation of the polyphagous mechanism selecting for prudence, as being reported for invasive alien consumers. For detailed explanations of signatures and how they were observed, see text

Invasion event	Signature				Key references
	1. Fast growth	2. Resource extirpation	3. Adjustment	4. Disappearance	
Comb jellyfish (*Mnemiopsis leidyi*) in the Black Sea	Yes	Yes	Yes	Decline	Kideys (2002)
Indo‐Pacific lionfish (*Pterois volitans*/*miles*) in Gulf of Mexico	Yes	Yes	Yes	Decline	Côté & Smith (2018)
					Harris et al. (2020)
Signal crayfish (*Pacifastacus leniusculus*) in Swedish lakes	Yes	Yes	Yes	Yes	Sandström et al. (2014)
					Ruokonen et al. (2014)
Argentine ant (*Linepithema humile*) in New Zealand	Yes	Yes	?	Yes	Cooling et al. (2012)
					Tillberg et al. (2007)

#### Comb jellyfish in the Black Sea

The comb jelly *Mnemiopsis leidyi* is a “voracious zooplanktonic predator” (Kideys, 2002), known to depress both abundance and diversity of mezoplankton (Fiori et al., 2019; Shiganova, 1998). After arriving in the Black Sea through ballast water, its outbreak in 1989 (with density $>1\;\mathrm{kg}\;m^{‐2}$) led to a sharp decline of anchovies, previously the dominating planktivores in the Black Sea, a result of both resource competition and predation on larvae (Kideys, 2002). Over the subsequent three years, however, *Mnemiopsis* declined about fivefold and anchovy catches recovered to their previous levels. Between 1992 and 1998, *Mnemiopsis* then coexisted with anchovy at this lower abundance (Kideys et al., 2000). Invasion of the predatory ctenophore *Beroe* (*B*. *ovata* or *B*. *cucumis*) in 1997 led to a further sharp decline of *Mnemiopsis* in 1999. Because *Beroe* feeds almost exclusively on *Mnemiopsis* (Finenko et al., 2001), it cannot entirely extirpate its prey. Currently, the two jellyfish therefore appear to persist in the Black Sea at low abundance.

#### Lionfish in the Gulf of Mexico

Indo‐Pacific lionfish *Pterois volitans* / *miles* grow and reproduces fast (Côté & Smith, 2018), deter predators with venomous spines (Côté & Smith, 2018; Vetrano et al., 2002), have high physiological tolerance and are effective predators, as they appear inconspicuous to their prey (Lönnstedt & McCormick, 2013). The course of their invasion of the Northern Gulf of Mexico and neighbouring areas since 1985 is exceptionally well studied (Côté & Smith, 2018; Harris et al., 2020). Prey extirpation by lionfish has been documented in controlled field experiments (Ingeman, 2016).

In 2018, Côté & Smith found first indications that the worst‐case scenario of lionfish invasion envisioned by Albins and Hixon (2013), “in which most reef‐fish biomass is converted to lionfish biomass, leaving invaded reefs depauperate of native fishes”, would not materialise. Benkwitt et al. (2017) reported for 64 unmanaged and unfished reefs in the Bahamas that lionfish populations first rapidly increased (70.6% per year), plateaued for between 2 and >7 years, and then, in some case, their unexplained declines (by up to 99% over a 4‐year period). Populations of the Nassau grouper (*Epinephelus striatus*), a comparable native predator, varied much less. Similarly, Harris et al. (2020) detailed what they called “precipitous declines” of lionfish populations in the Northern Gulf of Mexico over the period 2017–2019 (by up to 77–79%). Harris et al. associated this decline with an ulcerative skin disease observed on lionfish, but since this peaked in 2017 while the decline continued into 2019, other factors might also play a role.

#### Signal crayfish in Swedish lakes

Sandström et al. (2014) documented 40 years of population dynamics of North American signal crayfish (*Pacifastacus leniusculus*) that were introduced into 44 Swedish lakes. Most populations exhibited the rapid increase characteristic of invasive species, after which populations sizes stabilised. Yet, 41% of these populations collapsed after an average of 10.8 years without recovering. The authors considered and dismissed presence of predatory eel (*Anguilla anguilla*) and of crayfish plague (*Aphanomyces astaci*) to explain the collapses. Instead, they found subtle statistical effects of temperature and year of stocking. Based on evidence of strong density dependence in population time series and because it is known from Finnish lakes that *P*. *leniusculus* modifies and depauperates its macroinvertebrate prey community (Ruokonen et al., 2014), Sandström et al. (2014) offer resource overexploitation as a likely mechanistic explanation of the collapses.

#### Argentine ants in New Zealand

An example from the terrestrial realm is provided by populations of the Argentine ant (*Linepithema humile*) in New Zealand (Cooling et al., 2012). In the words of Cooling et al., “Introduced populations form high‐density, widespread, highly aggressive, unicolonial populations and can deleteriously influence native communities (Holway et al., 2002)”. While collapse and extirpation of invasive ant populations are common phenomena (Lester & Gruber, 2016), attribution of mechanisms can be hampered by insufficient understanding of ant diet and feeding behaviour. As Holway et al. (2002) point out, predation and scavenging must be distinguished. Noteworthy are therefore observations by Tillberg et al. (2007) that the trophic position of invading *L*. *humile* is highest at the invasion front and declines with the duration of site occupation, falling well below the trophic position of *L*. *humile* in its native range. This evidences resource depletion through predation that scavenging cannot explain.

Studying 150 sites with recorded *L*. *humile* presence in New Zealand, Cooling et al. (2012) found that 40% of populations had disappeared, with survival time in the rage of 10–18 years. Of the remaining populations, “many had shrunk from numerous nests covering multiple hectares with extremely high abundances to just one or two nests covering a very small area with low worker densities”. At infested sites, richness and abundance of other ant species was depressed but recovered after *L*. *humile* collapsed, providing additional indirect evidence of severe resource depletion by *L*. *humile*.

### Evidence of manifest prudence

From laboratory measurements of attack rate $a_{\mathrm{jk}}$ for preferred resources j of a consumer k, its assimilation efficiency ε, and respiration + mortality rate $\rho_{k}$ one can determine the minimum resource biomass $\rho_{k}/\left(\varepsilon a_{\mathrm{jk}}\right)$ or, in practice, biomass density that k requires to sustain its population. When this is similar to the resource density in k’s native habitat, we call this *manifest prudence*; it is the outcome predicted by our theory. If native resource density is much higher, a mechanism different from what we propose must be enabling consumer–resource coexistence. In cases where comparisons of absolute values of minimum required and native resource density are not possible, one can test for proportionality of the two quantities across contrasting groups of organisms.

#### Trends across ocean biogeographic regions

In pelagic ecology, $a_{\mathrm{jk}}=m_{k}^{‐1}s_{\mathrm{jk}}$ is called the *maximum specific clearance rate* and determined from consumer body mass $m_{k}$ and the maximum slope $s_{\mathrm{jk}}$ (dimension Volume/Time) or similar of a measured functional response. Marine ecologists have often studied whether pelagic consumers are food limited, and the question to what extend food is sufficient for survival got addressed along the way. In this context, Huntley and Boyd (1984) introduced $C_{m}=\rho_{k}/\left(\varepsilon a_{\mathrm{jk}}\right)$ as the ‘maintenance food concentration’. The contribution of mortality to $\rho_{k}$ is not usually considered, thus underestimating the true minimum requirement. Despite this, native resource densities tend to be lower than $C_{m}$ (Hirst & Bunker, 2003; Huntley, 1992; Mullin & Brooks, 1976)!

To reconcile this discrepancy, it has been argued that pelagic consumers might be able to access higher than average resource density, since resource distribution is patchy on the relevant scales (Huntley, 1992; Mullin & Brooks, 1976). Whatever the explanation, an abundance of data suggest that clearance or attack rates of marine pelagic consumers are not much higher than required to sustain their populations. Marine pelagic consumers are manifestly prudent. In particular, Huntley and Boyd (1984) showed that $C_{m}$ co‐varies with the variation in open‐water food availability for herbivorous marine zooplankton along the global temperature gradient.

Remarkable is also a meta‐analysis by Kiørboe (2011) showing that the geometric mean specific clearance rate of freshwater cladocerans (water fleas) is lower by an approximate factor 10 than that of marine copepods (which occupy a similar ecological niche). This is the trend expected from prudence, because in freshwater nutrients and food tend to be more abundant (Rossberg et al., 2019).

#### Trends across life forms

Kiørboe and Hirst (2014) conducted a meta‐analysis of respiration $\rho_{k}$ and specific clearance rates $a_{\mathrm{jk}}$ of marine pelagic species spanning a factor $10^{15}$ in body mass $m_{k}$. *Within* major taxonomic and life‐form groups (flagellates, ciliates, calanoid copeopods, non‐calanoid copeopods, euphasids, cnidaria and ctenophores, tunicates, pisces), they found both rates to scale as $m_{k}^{‐1/4}$. The lead coefficients (‘intercepts’) of these power laws, however, differed between taxonomic groups such that, when evaluated *across* all groups and body sizes, respiration and specific clearance rate both scaled approximately as $m_{k}^{0}$. Hence the changes between life forms in the lead coefficients for $\rho_{k}$ and for $a_{\mathrm{jk}}$ were such that $\rho_{k}/\left(\varepsilon a_{\mathrm{jk}}\right)$ remained similar across life forms. Kiørboe and Hirst (2014) were surprised by this result.

To see how prudence might explain this, note that in marine pelagic ecosystems biomass is approximately evenly distributed over the logarithmic body size axis (Sheldon et al., 1972), implying an approximately equal density of food available to organisms of all sizes. To be precise, biomass slightly declines with body mass (Rossberg et al., 2019), but so does species richness. The two effects plausibly compensate each other such that the biomass density of a consumer’s preferred resources is independent of consumer body mass. Manifest prudence then means invariance of $\rho_{k}/\left(\varepsilon a_{\mathrm{jk}}\right)$ across body size and life forms, as documented by Kiørboe and Hirst (2014).

Remarkably, the variation of $\rho_{k}$ around the overall geometric mean ($\approx 0.05\;\left(\mathrm{gC}/\mathrm{gC}\right)\;\mathrm{da}y^{‐1}$) found by Kiørboe and Hirst (2014) is smaller than that of $a_{\mathrm{jk}}$, and this mean value of $\rho_{k}$ is observed similarly across all domains of life (Makarieva et al., 2008). This agrees with our expectation (see section ‘Prudence and optimisation’) that $\rho_{k}$ will be near the physiological limit while $a_{\mathrm{jk}}$ is adjusted for prudence.

## PRUDENT PREDATION—THE WAY FORWARD

Both the theoretical and the empirical pictures we have drawn of the polyphagous mechanism for the evolution of prudence remain incomplete. Our theory represents several elements implicitly, including the metacommunity (O’Sullivan et al., 2021a), continuity of space (Goodnight et al., 2008), trophic trait matching (Appendix S1) and evolution on the generational timescale (Mitteldorf et al., 2002). Simulations making these elements explicit would be challenging but feasible, and useful for confirming their interaction in the ways we predict. In addition, while we presented empirical evidence of predicted processes (see section ‘Evidence of operation of the polyphagous selection mechanism’) and outcomes (sections ‘Evidence of ecological constraints on basic reproduction number’ and ‘Evidence of manifest prudence’), different evidence related to different systems. Future research should address these gaps.

What gives us confidence in the theory despite these caveats are its reliance on generic ecological principles and its tremendous explanatory power. All three specific patterns it predicts (preferred steepness values, delayed decline of invasive alien consumers, manifest prudence) have long been noticed but remained hitherto unexplained. Evolved prudence offers explanations for all three apparently unrelated loose ends or, in the words of Kuhn (1962), ‘anomalies’. A dismissal of evolved prudent predation would not only reopen the old question of how consumers and resources coexist in nature, it would also forfeit its potential for theoretical unification.

To both sceptics and enthusiasts of our theory we suggest more wide‐ranging testing for the predicted patterns across biota. For example, the analysis by Kiørboe and Hirst (2014) discussed in the section ‘Trends across life forms’ could be expanded to include biogeography (see ‘Trends across ocean biogeographic regions’), and databases such as FoRAGE (DeLong & Uiterwaal, 2018) might permit its extension beyond marine pelagic systems.

## AUTHOR CONTRIBUTIONS

OUGAK and AGR jointly conceived the study, conceived and developed the metapopulation model, and wrote the manuscript. OUGAK conceived and implemented the deconstructed model formulation and the simplified model for consumer impact, compiled evidence on invasive alien species, and performed model simulations and model analyses. AGR developed the analytic theory underlying Equation (10), implemented the full model formulation and compiled most evidence from the empirical literature.

### PEER REVIEW

The peer review history for this article is available at https://publons.com/publon/10.1111/ele.13979.

## Supporting information

Supplementary Material

## Data Availability

This study did not generate new data.
