# Supplementary material for: Evolution of prudent predation in complex food webs
Source: Ecol Lett. 2022 Mar 1;25(5):1055–74. doi: 10.1111/ele.13979 (PMC9540554; doi:10.1111/ele.13979)
Supplement: Supplementary file 1 — Supplementary Material [file ELE-25-1055-s001.pdf]

Supporting Information for Evolution of prudent predation in complex food webs: mechanism and evidence

Orestes Uxio Gutierrez Al-Khudhairy and Axel G. Rossberg

January 13, 2022

Appendix S1 Motivation of our algorithm for sampling species

We explain here in detail why Eqs. (3) and (4) are plausible approximations for sampling the interaction strengths of new species entering our model community.

Under quite general conditions it is possible to approximate the dependence of attack rates on the traits of consumers and resources in the form (Rossberg *et al.*, 2010; Nagelkerke & Rossberg, 2014; Rossberg, 2013, Ch. 8):

$$a_{jk} \approx a_0 \exp\left[v_0^{(j)} + f_0^{(k)} - \sum_{k=1}^{D} \frac{\sigma_k}{2}\left(v_k^{(j)} - f_k^{(k)}\right)^2\right], \tag{S1}$$

with D denoting the dimensionality of trophic niche space and $v_0^{(j)}, \dots, v_D^{(j)}$ and $f_0^{(k)}, \dots, f_D^{(k)}$ *vulnerability-* and *foraging traits* of resources and consumers, respectively. These *trophic traits* can be computed as functions of observable biological traits (Nagelkerke & Rossberg, 2014). A similar representation has been proposed by Rohr *et al.* (2010). The constant a_0 has dimensions of attack rates and $\sigma_k = \pm 1$. There is some ambiguity in how to choose a_0, σ_k and the functions mapping observed traits to trophic traits. However, when imposing a condition that the mean of $(v_0^{(j)})^2$ over the entire resource pool j is minimised, these ambiguities are resolved up to rigid geometric transformations of the vectors $\mathbf{v}^{(j)} = (v_1^{(j)}, \dots, v_D^{(j)})$ and $\mathbf{f}^{(k)} = (f_1^{(j)}, \dots, f_D^{(j)})$ (Rossberg, 2013, Ch. 8) and the choice of a_0. With the mean of $(v_0^{(j)})^2$ thus minimised, we shall approximate $v_0^{(j)} = 0$.

For large D and sufficient statistical independence of the components of $\mathbf{v}^{(j)}$ and $\mathbf{f}^{(k)}$ (Rossberg, 2013, Ch. 11), one can approximate the sum in Eq. (S1) for randomly sampled consumer-resource pairs (j, k) by a normal distribution. Denoting the mean of this normal distribution by μ, its variance by σ^2, and defining $a_k = a_0 \exp(f_0^{(k)} - \mu)$, this leads to Eq. (4).

All traits of consumers and resources can undergo mutations. However, compared to the evolution of foraging traits $f_0^{(k)}, \dots, f_D^{(k)}$, the resulting evolution of vulnerability traits $v_0^{(j)}, \dots, v_D^{(j)}$ is known to be slow (Rossberg *et al.*, 2006; Bersier & Kehrli, 2008; Eklöf & Stouffer, 2016)—a median of 25 times slower in an analysis of Rossberg *et al.* (2006). It shall here be disregarded.

Mutations of any observable biological traits will affect several foraging traits $f_0^{(k)}, \dots, f_D^{(k)}$. The question whether this increases of decreases *short-term fitness* (Goodnight *et al.*, 2008) in a given community depends not only on all traits $f_0^{(k)}, \dots, f_D^{(k)}$ of the focal consumer k but also on the sets of resources and competitors in the community. Even when a mutation leads to an increase in short-term fitness, the change in $f_0^{(k)}$ associated with this mutation might be positive or negative, because, provided niche space dimensionality D is not too low, the associated change in $f_0^{(k)}$ is just one of many random changes in foraging traits resulting from this mutation. Mutants arriving at the focal patch from a source patch may therefore have $f_0^{(k)}$ values that can be higher or lower than the $f_0^{(k)}$ of the propagule that founded the population in the source patch. Because, all else equal, smaller $f_0^{(k)}$ correspond to consumers that, overall, forage less efficiently than consumers with larger $f_0^{(k)}$, and low efficiency is mechanically easier to achieve than high efficiency, one must plausibly assume that degeneration of traits through

mutations (Pomiankowski *et al.*, 1991) leads to a decay of $f_0^{(k)}$ on average unless this is counteracted by selection pressure. Recalling that $a_k = a_0 \exp(f_0^{(k)} - \mu)$, this leads to Eq. (3).

We assume that the relevant species pools are large and diverse, such that different patches have in effect statistically independent, typically non-overlapping species compositions. The random variables ξ_{jk} in Eq. (4) are therefore sampled anew as a propagule arrives at the focal patch, independent of a consumer's interactions with the residents of its source patch. Only the inheritance of a_k must be accounted of.

As a caveat, we note that in reality vulnerability traits do not cover the D-dimensional trophic traits space evenly, e.g. because these traits carry phylogenetic signal (related species have similar consumers, Bersier & Kehrli 2008). Then foraging traits other than $f_0^{(k)}$ might contribute to long-term fitness as well. For simplicity, we disregard this complication in our model.

Appendix S2 Derivation of the sub-models of the deconstructed formulation

We provide the rationale and outline the derivation of the four criteria Eqs. (13-17) driving invasions and extirpations in the deconstructed model formulation.

The *invasibility criterion*, Eq. (13), predicts invasibility when disregarding the presence of all but the focal consumer in the dimensionless full model, Eq. (2). Formally, it is obtained by computing the equilibrium state of Eq. (2) for $S_{\mathrm{C}} = 1$ and $B_k^{\mathrm{C}} = 0$ (with $k = 1$), which is $B_j^{\mathrm{R}} = 1$ for $1 \leq j \leq S_{\mathrm{R}}$, and then extracting the condition that, by Eq. (2b), this equilibrium is unstable such that the consumer can invade: $\sum_{j=1}^{S_{\mathrm{R}}} H_{jk} B_j^{\mathrm{R}} - 1 = \sum_{j=1}^{S_{\mathrm{R}}} H_{jk} - 1 > 0$.

The condition for the *overexploitation* of resource j during the expansion phase of an invading consumer k, Eq. (15), is obtained by analysing the dimensionless full model, Eq. (2), for the case of only one consumer and one resource: $S_{\mathrm{C}} = 1$, $S_{\mathrm{R}} = 1$ (with $j = k = 1$). We consider again the situation where the consumer is initially absent $B_k^{\mathrm{C}} = 0$ and the resource at equilibrium $B_j^{\mathrm{R}} = 1$, $dB_j^{\mathrm{R}}/dt = 0$. Then the consumer invades at low abundance. To estimate the minimum of B_j^{R} attained during the consumer invasion, i.e. during the transient before a new equilibrium is reached, we approximate dynamics by disregarding the density dependence of resource production expressed by the term $-B_j^{\mathrm{R}}$ in Eq. (2a). This approximation is justified because we are interested in situations where B_j^{R} falls below $M_{\min} \ll 1$. It reduces the model to the classical Lotka-Volterra predator-prey equations

$$\frac{dB_j^{\mathrm{R}}}{dt} = r \left[1 - H_{jk} B_k^{\mathrm{C}} \right] B_j^{\mathrm{R}}, \tag{S2a}$$

$$\frac{dB_k^{\mathrm{C}}}{dt} = \rho_k \left[H_{jk} B_j^{\mathrm{R}} - 1 \right] B_k^{\mathrm{C}}. \tag{S2b}$$

Evaluating the conservation law known for this system (Lotka, 1920) for the initial conditions $B_j^{\mathrm{R}} = 1$, $dB_j^{\mathrm{R}}/dt = 0$, one finds that at its minimum B_j^{R} satisfies $\ln(B_j^{\mathrm{R}}) = -H_{jk}(1 - B_j^{\mathrm{R}})$ (Rossberg, 2013, Sec. 20.3.3). Since we are interested in situations where the minimum is deep ($B_j^{\mathrm{R}} < M_{\min} \ll 1$), this condition can be approximated as $\ln(B_j^{\mathrm{R}}) = -H_{jk}$. It follows that B_j^{R} falls below $M_{\min}$ during consumer k's invasion if $\ln(M_{\min}) > -H_{jk}$, which is equivalent to Eq. (15).

The conditions for *consumer-mediated competitive exclusion*, for *exploitative competitive exclusion* and for *Pyrrhic competition* all derive directly from exact equilibrium solutions of the dynamic model. The general multispecies model, Eq. (2), is well studied (MacArthur, 1970, 1972; Case & Casten, 1979; Chesson, 1990). To write down its equilibrium solution, let $\mathbf{H}$ be the matrix with entries H_{jk} and define the competition matrix as the matrix with entries

$$C_{kl} = \sum_{j=1}^{S_{\mathrm{R}}} H_{jk} H_{jl}, \quad \text{that is} \quad \mathbf{C} = \mathbf{H}^T \mathbf{H}. \tag{S3}$$

Denote by $\mathbf{s}$ the vector of intrinsic consumer growth rates

$$s_k = R_k - 1, \tag{S4}$$

with $R_k = \sum_{j=1}^{S_{\mathrm{R}}} H_{jk}$ defined as in the main text. The vector $\mathbf{b}^{\mathrm{C}}$ of consumer population biomasses B_j^{C} at equilibrium is then given by

$$\mathbf{b}^{\mathrm{C}} = \mathbf{C}^{-1} \mathbf{s}. \tag{S5}$$

and that of resource population biomasses B_j^{R} by

$$\mathbf{b}^{\mathrm{R}} = 1 - \mathbf{H} \mathbf{B}^{\mathrm{C}}. \tag{S6}$$

In the case of only one consumer ($S_{\mathrm{C}} = 1$, $k = 1$), the biomass of the resource j is therefore $B_j^{\mathrm{R}} = 1 - H_{jk}(C_{kk})^{-1} s_k$. The resource with the lowest biomass is that with the largest H_{jk}, i.e., the main resource of k. Its biomass is negative, implying resource extinction (Holt, 1977), if

$$C_{kk} < H_{jk} s_k. \tag{S7}$$

The criterion for consumer-mediated competitive exclusion, Eq. (16), spells out this condition.

For the two-consumer ($S_c = 2$) problem, we have, with $k = 1$ and $l = 2$,

$$\mathbf{C}^{-1} = \frac{1}{C_{kk}C_{ll} - C_{kl}^2} \begin{pmatrix} C_{ll} & -C_{kl} \\ -C_{kl} & C_{kk} \end{pmatrix}. \tag{S8}$$

Combining Eqs. (S5) and (S8), we find that (for $S_c = 2$) $B_k^C < 0$ if

$$C_{ll}s_k - C_{kl}s_l < 0 \tag{S9}$$

or equivalently

$$s_k < \frac{C_{kl}s_l}{C_{ll}}. \tag{S10}$$

Our criterion of exploitative competitive exclusion, Eq. (14) spells out this condition.

Now, assume that Eq. (S10) and the corresponding condition with l's and k's role reversed both fail to be satisfied. This alone does not guarantee coexistence of all species. Combining Eqs. (S5), (S6) and (S8), one can see that the equilibrium abundance of resource B_i^{R} is predicted to be negative if

$$1 < H_{ik}\frac{C_{ll}s_k - C_{kl}s_l}{C_{kk}C_{ll} - C_{kl}^2} + H_{il}\frac{C_{kk}s_l - C_{kl}s_k}{C_{kk}C_{ll} - C_{kl}^2}. \tag{S11}$$

This can be re-arranged to

$$C_{kk}C_{ll} - C_{kl}^2 < s_k\left(H_{ik}C_{ll} - H_{il}C_{kl}\right) + s_l\left(H_{il}C_{kk} - H_{ik}C_{kl}\right), \tag{S12}$$

and our condition for Pyrrhic competition, Eq. (17), spells out this inequality.

We now outline how these conditions can efficiently be evaluated for large S_{R} and S_{C}. The most time-consuming step is the computation of $\mathbf{C}$ in Eq. (S3), as (for practical purposes) the number of operations this requires increases as $\mathcal{O}(S_{\mathrm{C}}^2 S_{\mathrm{R}})$ with system size. All remaining calculations can be done using just $\mathcal{O}(S_{\mathrm{C}}^2)$ or $\mathcal{O}(S_{\mathrm{C}}S_{\mathrm{R}})$ operations.

Denote, for any square matrix A, by $\mathbf{diag}(A)$ the vector formed by its diagonal elements, and by $\mathbf{Diag}(\mathbf{v})$, for any vector $\mathbf{v}$, the diagonal matrix with $\mathbf{v}$ on the diagonal. We can evaluate the $S_{\mathrm{C}} \times S_{\mathrm{C}}$ matrix $\mathbf{\Phi}$ with entries Φ_{kl} given by the left-hand side of Eq. (S9) as

$$\mathbf{\Phi} = \mathbf{s}\,\mathbf{diag}(\mathbf{C})^T - \mathbf{C}\,\mathbf{Diag}(\mathbf{s}). \tag{S13}$$

To test for extirpations, set the diagonal of $\mathbf{\Phi}$ to exactly zero to remove small numerical errors. Extirpation of consumer k by our (simplified) criterion follows if row k of $\mathbf{\Phi}$ constrains negative elements.

The $S_{\mathrm{C}} \times S_{\mathrm{C}}$ matrix $\mathbf{D}$ with entries $D_{kl} = C_{kk}C_{ll} - C_{kl}^2$, containing the determinants of all two-consumer competition problems (the denominators in Eqs. (S8), (S11)), can be computed as

$$\mathbf{D} = \mathbf{diag}(\mathbf{C})\,\mathbf{diag}(\mathbf{C})^T - \mathbf{C} \circ \mathbf{C}, \tag{S14}$$

with $\circ$ denoting elementwise multiplication. After finding for each consumer k the index $m(k)$ of its main resource, one can constructed the $S_{\mathrm{C}} \times S_{\mathrm{C}}$ matrix $\mathbf{M}$ with entries

$$M_{kl} = H_{m(k)l}. \tag{S15}$$

Using this, we obtain the $S_{\mathrm{C}} \times S_{\mathrm{C}}$ matrix $\mathbf{\Delta}$ with entries given by the difference between left- and right-hand side of Eq. (S12) for the main resource of each consumer k as

$$\mathbf{\Delta} = \mathbf{D} - \mathbf{Diag}(\mathbf{diag}(\mathbf{M}))\mathbf{\Phi} - \mathbf{M} \circ \mathbf{\Phi}^T. \tag{S16}$$

To test for extirpations, set the diagonal of $\mathbf{\Delta}$ to exactly zero to remove small numerical errors. Extirpation of the main resource of consumer k by our (simplified) criterion follows if row k of $\mathbf{\Delta}$ contains negative elements.

By striking a new balance between code complexity, speed, and accuracy in the multi-objective optimisation problem of finding fast, simple and accurate models, our deconstructed formulation carves out emergent properties (*sensu* Rossberg, 2007) of the full model, Eq. (2), e.g., those shown in Figs. 2 and 3.

Appendix S3 Evolutionary steady-state condition including mutation bias

We derive the steady state condition for base attack rate, Eq. (6).

To understand the effect of mutation bias, we invoke the Price equation (Price, 1972). It predicts that the expected rate of evolutionary change of a trait q of a species is given by

$$d\mathsf{E}q/dt = \mathrm{cov}[f(q), q] + \mathsf{E}\dot{q}, \tag{S17}$$

with $f(q)$ denoting the invasion fitness (for a given environment) of lineages of type q, and the last term representing the mutation bias (the mean inherent rate of change of traits). For trait values q^* corresponding to evolutionary steady states, both sides of Eq. (S17) must evaluate to zero. Following Page & Nowak (2002), we expand $f(q)$ to first order at $q = q^*$. Combined with the population-dynamical equilibrium condition $f(q^*) = 0$, this leads to $0 = f'(q^*)\,\mathrm{var}\,q + \mathsf{E}\dot{q}$, or equivalently

$$f'(q^*) = -\frac{\mathsf{E}\dot{q}}{\mathrm{var}\,q}. \tag{S18}$$

This condition generalises the conventional criterion for evolutionary singular strategies, $f'(q^*) = 0$, to situations with mutation bias.

To apply this result to our model, we first note that our way of implementing evolution of base attack rate in our model through Eq. (3) makes the scheme formally analogous to how individual-based models describe evolution of a trait in a population. In this analogy, (i) coexisting populations of consumers in our model correspond to individuals in conventional models, (ii) mutations accumulated between successive invasion events along lineages in our model correspond to mutations between successive generations in conventional models, and (iii) the community of consumers at the focal patch in our model corresponds to the evolving population in conventional models.

While this formal analogy does not represent the process our community assembly model actually describes, it allows us to use Eq. (S18), with $q = \ln a$, to obtain the steady-state condition for mean logarithmic base attack rate in this model. With the assumed statistical equivalence of all patches in the metacommunity (mean-field approximation), such a steady state can arise only if the evolution of the base attack rates of all consumers in the metacommunity has reached a steady state. The steady state condition for the community mean of $\ln a$ therefore implies a steady-state condition for the evolution of $\ln a_k$ for all species k in the metacommunity (see also Appendix S6).

To evaluate Eq. (S18) with $q = \ln a$, we set

$$\mathsf{E}\dot{q} = \frac{\ln \gamma_0}{L^*}, \tag{S19}$$

where L^* is the mean lifetime of populations in the community. The standing mutational variance $\mathrm{var}\,q = \mathrm{var}(\ln a)$ is obtained from the distribution of a over the simulation steady state.

We approximate steady-state invasion fitness, i.e. the mean intrinsic rate of increase ($f(q) > 0$) or decrease ($f(q) < 0$) of the number of populations of type q in the simulation steady state, as $f(q) \approx \ln[R(a)]/L(a)$, where $R(a)$ and $L(a)$ are as defined in the main text. With a^* representing the geometric mean of a over the simulation steady state, such that $\ln a^*$ is the arithmetic mean of $\ln a$, we expect that $R(a^*) = 1$. This leads to

$$f'(q^*) \approx \left.\frac{d\{\ln[R(a)]/L(a)\}}{d\ln a}\right|_{a=a^*} = \left.\frac{1}{L(a)}\frac{d\ln[R(a)]}{d\ln a}\right|_{a=a^*} \approx \left.\frac{1}{L^*}\frac{d\ln[R(a)]}{d\ln a}\right|_{a=a^*}. \tag{S20}$$

Putting Eqs. (S19) and (S20) into Eq. (S18) and multiplying both sides with L^* gives

$$\left.\frac{d\ln R(a)}{d\ln a}\right|_{a=a^*} \approx -\frac{\ln\gamma_0}{\mathrm{var}(\ln a)}. \tag{S21}$$

Applying the identity $\ln(x) = \log_{10}(x)/\log_{10}[\exp(1)]$ yields Eq. (6).

Appendix S4 Mechanisms determining 'birth rate'

Within the mean-field approximation, the 'birth rate' function $b(a)$ is in our model the rate at which resident populations with base attack rate a give rise to successful invaders into the model community *via* Eq. (3). To derive an analytic approximation of $b(a)$, we must account for three model elements, which are common to both model formulations:

1. The mutation step, Eq. (3), determining the new consumer's base attack rate from that of the resident.

2. The sampling of the new consumer's attack rates according to Eq. (4), and the test whether it can invade.

3. The fact that time is measured in numbers of successful consumer invasions.

Crucial is the probability of successful invasion in 2. We begin with an analysis of this element, adding subsequently considerations of 1 and 3.

We first consider the deconstructed model formulation. On one hand, competitive exclusion by a resident consumer according to Eq. (14) of the algorithm always implies an inability to invade according to Eq. (13), so that (for $S_{\mathrm{C}} > 0$) only Eq. (14) needs to be considered. On the other hand, Eq. (14) can be read as just a correction of the invasibility criterion, Eq. (13) to account for the presence of competitors. To see this, re-arrange Eq. (14) as

$$\sum_{j=1}^{S_{\mathrm{R}}} H_{jk} \left[1 - H_{jl} \frac{\sum_{i=1}^{S_{\mathrm{R}}} H_{il} - 1}{\sum_{i=1}^{S_{\mathrm{R}}} H_{il}^2} \right] - 1 < 0. \tag{S22}$$

The term in square brackets represents the population biomass (in units of K) that resource j would have if l was the only extant consumer. The deconstructed formulation ensures that, at the end of a model iteration, no extant resource satisfies the criterion for consumer-mediated competitive exclusion, Eq. (16) and all extant consumers satisfy the simple invasibility criterion, Eq. (13). These loop invariants guarantee that the value of the expression in brackets in Eq. (S22) lies between 0 and 1 for all k and l. Satisfaction of Eq. (S22) therefore implies violation of Eq. (13).

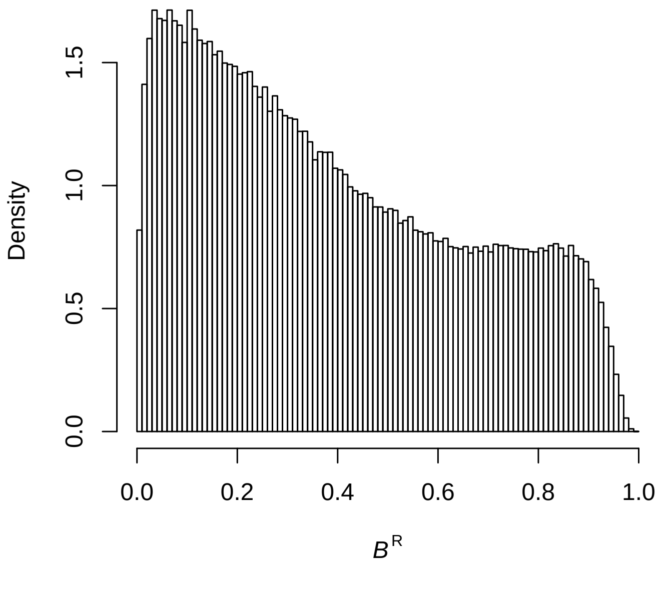

Figure S1: Histogram of resource biomasses B^{R} in the steady state of the full model, sampled from community snapshots after every 200 consumer additions. Neither values close to zero nor values close to one are very frequent.

Because there is no mechanism active in the model that would favours values of the expression in square brackets above that are particularly close to zero (see also Fig. S1), most of the variation among the addends in the sum over j in Eq. (S22) is due to the log-normal distribution of the invader's attack rates H_{jk}. The presence of competitors merely moderates the effect of this variation. It can therefore be approximated by substituting the square bracket by a suitable constant $0 < \beta < 1$: the fitting parameter introduced in the main text.

The sum over j in Eq. (S22) can then be written as $\alpha_0 a_k \beta \sum_{j=1}^{S_R} e^{\sigma \xi_{jk}}$. The distribution of the sum in this last expression is, for a given number of resources S_R, often well approximated by a single log-normal distribution with suitable choices for mean $\mu_{S_R} \approx \sigma\sqrt{2 \ln S_R}$ and standard deviation $\sigma_{S_R} \approx \sigma/\sqrt{1 + 2 \ln S_R}$ of the logarithm (Rossberg *et al.*, 2011). (We estimated μ_{S_R} and σ_{S_R} numerically form 10,000 samples of log-normal sums, which is more accurate.)

From this log-normal approximation, the invasion probability for species with given base attack rate a_k is obtained as

$$P_{\mathrm{inv}}(a_k) = \Phi\left(\frac{\ln(\alpha_0 \beta a_k) + \mu_{S_R}}{\sigma_{S_R}}\right), \tag{S23}$$

with $\Phi(x)$ denoting the cumulative standard normal distribution function. For the full model, the same functional form as in Eq. (S23) can be chosen based on the same rationale: compared to the variation in link strengths, the variation among the population biomasses of resident resources is small.

Denote by $P^*_{\mathrm{inv}}(a)$ the probability that the "offspring" of a resident species with base attack rate a can invade the community. The log-normal approximation for the sum in $\alpha_0 a_k \beta \sum_{j=1}^{S_R} e^{\sigma \xi_{jk}}$ used above combines seamlessly with the log-normal distribution of a_k resulting from the mutation of base attack rate a_l of the 'parent' population l as per Eq. (3). We can therefore obtain $P^*_{\mathrm{inv}}(a)$ from Eq. (S23) by correcting $\mu^*_{S_R} = \mu_{S_R} + \ln \gamma_0$ and $\sigma^*_{S_R} = [\sigma^2_{S_R} + (\ln \gamma_1)^2]^{1/2}$ to account for mutational variance and bias. Hence

$$P^*_{\mathrm{inv}}(a) = \Phi\left(\frac{\ln(\alpha_0 \beta a) + \mu^*_{S_R}}{\sigma^*_{S_R}}\right). \tag{S24}$$

Because we measure time in units of consumer invasions, and both variants of our model attempt consumer invasions until one succeeds, the probability for "offspring" of resident consumer l to invade in a given time step is $P^*_{\mathrm{inv}}(a_l)/\sum_{k=1}^{S_C} P^*_{\mathrm{inv}}(a_k)$ (guaranteeing that the probability for offspring of some consumer k to invade evaluates to 1). Since species richness and the distribution of the a_l fluctuate somewhat through time, we calculated the 'birth rate' in Fig. 3c,g as the average of this probability for a given base attack rate a over the model steady state:

$$b(a) = \text{Average through time of } \frac{P^*_{\mathrm{inv}}(a)}{\sum_{k=1}^{S_C} P^*_{\mathrm{inv}}(a_k)}. \tag{S25}$$

Appendix S5 Serial extinction

We derive Eq. (10), which predicts a consumer's intrinsic growth rate after serial extinction in the limit of high base attack rate and species richness.

Note first that, because during serial extinction resources are successively removed in decreasing order of the consumer's attack rate (and also in the simplified model, Box 2), the distribution of attack rates after serial extinction is the same as before, except for being truncated from above at the point where Eq. (16) gets violated. In situations where the sums in Eq. (16) are not dominated by just a few resources, the central limit theorem can be invoked and the sums approximated by their expectation values, which then permits analytic computation of the truncation threshold H_* and other properties of the end state.

The calculations simplify by first approximating the relevant section of the upper tail of the log-normal attack-rate distribution, Eq. (4), by a Pareto distribution, which can be derived in the limit of high resource richness S_R (Rossberg *et al.*, 2011; Rossberg, 2013). By this approximation, the consumer has on average Z resources with H_{jk} larger than some "observation threshold" H_0, and for these

$$P[H_{jk} \le x] \approx 1 - \left(\frac{H_0}{x}\right)^{\nu}, \tag{S26}$$

with $\nu = \sigma^{-1}\sqrt{2 \ln S_\mathrm{R}}$. Empirically, typical values for ν are in the range 0.5 to 0.6 (Rossberg *et al.*, 2011; Rossberg, 2013). Values $\nu \ge 1$ would correspond to extreme omnivory where the proportional contribution of each resource species to a consumer's diet scales as $1/S_\mathrm{R}$, i.e. no resource makes a sizeable contribution to the diet. We are unaware of such a situation occurring in nature, and therefore assume $0 < \nu < 1$ in this study.

For a given observation threshold H_0 one can define the consumer link density Z, i.e. the mean over all consumers of the number of resources with scaled attack rate H_{jk} above the threshold H_0. By choosing Z, we can control the typical strengths H_max of the strongest attack rate before serial extinction, specifically the $\exp(-1)$-quantile of the distribution of $\max_j H_{jk}$. In the limit of large Z, this leads to the condition

$$\exp(-1) = (P[H_{jk} \le H_\mathrm{max}])^Z = \left[1 - \left(\frac{H_0}{H_\mathrm{max}}\right)^{\nu}\right]^Z \approx \exp\left[-\left(\frac{H_0}{H_\mathrm{max}}\right)^{\nu} Z\right] \tag{S27}$$

and so

$$Z \approx \left(\frac{H_\mathrm{max}}{H_0}\right)^{\nu}. \tag{S28}$$

It goes without saying that H_max is proportional to base attack rate a_k and can therefore be use as a proxy for the latter.

With this preparation, we can now take expectation values on both sides of Eq. (16) for the case of truncation of the link-strength distribution at H_*, the largest value for which Eq. (16) is not violated. This leads to a condition

$$\mathsf{E}\left[\sum_j^{H_{jk}\le H_*} H_{jk} - 1\right] = H_*^{-1}\mathsf{E}\left[\sum_j^{H_{jk}\le H_*} H_{jk}^2\right], \tag{S29}$$

which can be written as

$$\left[Z\int_{H_0}^{H_*} p(x) x dx - 1\right] = H_*^{-1} Z \int_{H_0}^{H_*} p(x) x^2 dx, \tag{S30}$$

where $p(x) = -(d/dx)P[H_{jk} \le x]$ is the probability density of the untruncated attack rate distribution. Evaluation of the integrals after inserting Eq. (S26) leads to

$$\frac{Z\nu H_*^{-\nu}\left(H_* H_0^{\nu} - H_*^{\nu} H_0\right)}{1-\nu} - 1 = H_*^{-1}\frac{Z\nu H_*^{-\nu}\left(H_*^2 H_0^{\nu} - H_*^{\nu} H_0^2\right)}{2-\nu} \tag{S31}$$

and, after inserting Eq. (S28) and taking the limit of low observation threshold ($H_0 \to 0$),

$$\frac{H_*^{-\nu}\left[\nu H_* H_\mathrm{max}^{\nu} - H_*^{\nu}(1-\nu)\right]}{1-\nu} = \frac{\nu H_*^{1-\nu} H_\mathrm{max}^{\nu}}{2-\nu}. \tag{S32}$$

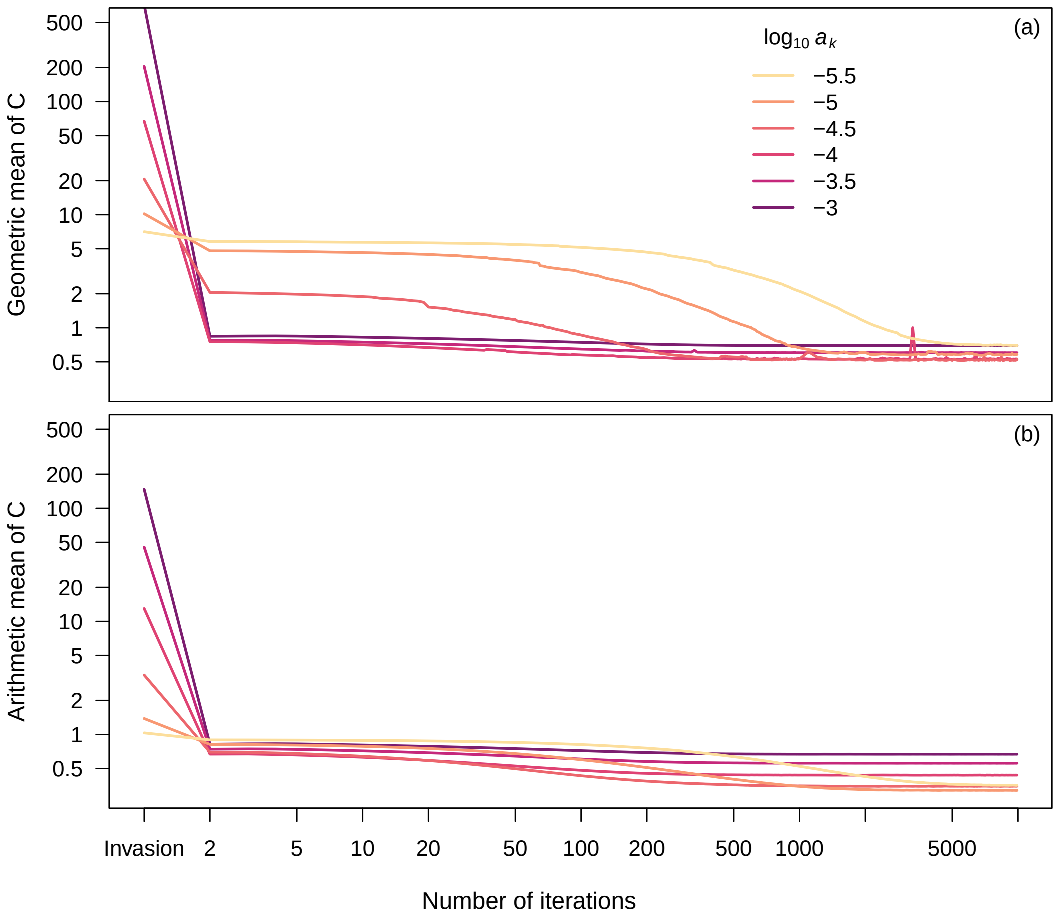

Figure S2: **Dependence of intrinsic growth rate term $\mathsf{C} = 1 - \sum_j H_{jk}$ on base attack rate in the course of repeated serial extinction and resource turnover.** Panel (a) shows geometric means of C over 10^6 replicated runs of the model of Box 2 over 10^4 iterations, panel (b) arithmetic means. For high base attack rates a_k (dark lines), both geometric and arithmetic means approach the same value ≈ 0.86 (indicating a near-deterministic outcome) after the first iteration of consumer-mediated competitive exclusion, largely independent of base attack rate, as predicted by the analytic theory. The value is different from the analytic prediction $1 - \sigma^{-1}\sqrt{2 \log S_\mathsf{R}} \approx 0.14$ valid for large because S_R, because $S_\mathsf{R} = 224$ is not sufficiently large.

This equation can be solved for H_*, yielding

$$H_* = \left[\frac{(1-\nu)(2-\nu)}{\nu H_{\max}^{\nu}} \right]^{1/(1-\nu)} . \tag{S33}$$

The expected intrinsic growth rate of the consumer after serial extinction equals the left-hand sides of Eqs. (S29)-(S32). When putting Eq. (S33) into the left-hand side of Eq. (S32) it simplifies considerably, leading to the final result

$$\mathsf{E} \left[\sum_k^{H_{ki} \le H_*} H_{ki} - 1 \right] = 1 - \nu. \tag{S34}$$

With $0 < \nu < 1$, this result implies that $\mathsf{E} \sum_j^{H_{jk} \le H_*} H_{jk}$ attains values between 1 and 2. On the other hand, the upper cutoff H_* declines with increasing $H_{\max}$ (or base attack rates a_i) as $H_{\max}^{-\nu/(1-\nu)}$ by Eq. (S33). For large base attack rates and so large $H_{\max}$, the sum $\sum_j^{H_{jk} \le H_*} H_{jk}$ therefore has contributions from many small terms, justifying our application of the central limit theorem to approximate the sums entering Eq. (16) by their expectation values. Figure S2b qualitatively confirms this result.

Interestingly, above considerations imply that, despite having the same niche width in terms of the spread σ of the log-normal attack-rate distribution, invaders with higher base attack rate will have more diverse diets post Impact than those with lower attack rates. This might explain why invasive alien consumers are often found to be 'generalists'.

Box S1 Algorithm of the evolutionary metapopulation model.

The model state is given by N patches which are either empty or occupied by a population with base attack rate a_i ($1 \leq i \leq N$). The model is simulated as follows:

1. Occupy a proportion p of patches with populations with identical initial base attack rates a_i.
2. Select an occupied source patch l for dispersal. Sample the base attack rate a_k of a propagule according to Eq. (3).
3. Sample a target patch j.
4. If patch j is occupied:
   (a) If $a_j < a_k$, replace the new population of patch j with one that has base attack rate a_k, otherwise do nothing.
5. If patch j is not occupied:
   (a) With invasion probability $P_{\mathsf{inv}}(a_k)$, establish in patch j a new population with base attack rates a_k and then remove the population from another occupied patch m, sampled at random from all occupied patches with probability proportional to $1/L(a_m)$. $P_{\mathsf{inv}}(a)$ is our approximation of invasion probability for the deconstructed community model, Eq. (S23) with $\beta = 0.45$ and $S_{\mathsf{R}} = 224$ (corresponding to the mean equilibrium richness in Fig. 2b), and $L(a)$ the polynomial fit to mean population lifetime in Fig. 3h ($\log_{10} L = -0.04105026(\log_{10} a)^2 - 0.78404937 \log_{10} a - 0.77341520$).
6. Continue from Step 2 for a predetermined number of iterations.

The values of γ_0, γ_1, and σ are as in Tab. 1.

The algorithm can be reformulated in such a way that only a list of the a_i value of occupied patches i is kept in memory. In each iteration, Step 4a is then executed with probability p and otherwise Step 5a. When invasion is successful in Step 5a, the new a_k value is stored in the memory location where a_m was previously stored. This formulation permits us to take the limit $p \to 0$ while keeping the number of occupied patches pN fixed.

Appendix S6 The limited impact of cheaters

Cheaters exploit benefits offered by more altruistic conspecifics to their advantage, thus potentially counteracting the evolution of altruism. To obtain a bound on the impact of cheaters on prudent predation, we devised a simple evolutionary metapopulation model. For a single species, this model explicitly describes the kind of evolutionary process that the evolutionary community model, introduced in the main text, implicitly describes for many species using a mean-field approximation. By comparing the evolutionary steady states reached by the two models for the special case where cheating is absent, the plausibility of the mean-field approximation can be tested.

Specifically, the evolutionary metapopulation model describes a landscape of N patches that are either occupied by the focal species or not. The population occupying patch i ($1 \leq i \leq N$) has an associated base attack rate a_i. We assume that cheating occurs if a population of the focal species disperses to a patch that is already occupied, and the propagule's base attack rate is larger than that of the resident in that patch. The propagule then replaces the resident population. This model disregards that conspecific propagules will not only differ in their base attack rates from residents, but also in other foraging traits (Appendix Appendix S1), and therefore have, on average, a reduced likelihood of encountering suitable resources and establishing themselves. Our metapopulation model is therefore biased to overestimate the likelihood of cheating. We shall see that the predicted impact of cheating remains limited despite this.

Contrasting conventional stochastic patch occupancy models in the tradition of Levins (1969), patch occupancy p, i.e., the proportion of occupied patches, is a parameter in our model. The reason is evidence that species richness both at patch level (α) and at landscape level (γ) is regulated through ecological structural stability limits (O'Sullivan *et al.*, 2019), which our metapopulation model cannot explicitly represent. Mean occupancy is uniquely determined by α and γ as $p = \alpha/\gamma$. By fixing p we represent these limits implicitly.

The model is detailed in Box S1. Crucially, the dependencies on a of invasion probability and of the mean lifetime of populations are chosen to reproduce those of the deconstructed formulation of our food-web model (Fig. 3). We chose $pN = 1000$ over a range of p values, evaluated the algorithm over $4 \cdot 10^7$ iterations, and sampled base attack rates from the last 3/4 of each run to characterise the steady state (which was reached after less than a 10th of iterations).

In the limit $p \to 0$, where cheating does not occur, the metapopulation model should recover the mean base attack rate in the evolutionary steady state of our food-web model. Indeed, the metapopulation model attained a steady state with mean logarithmic base attack rate $\overline{\log_{10} a} = -5.14$, close to the value of -4.96 obtained with the deconstructed model formulation. We also obtained an approximately normal distribution of $\log_{10} a$ in the steady state of the metapopulation model similar to that in Fig. 3e. These results provide evidence that our reconstruction of the fitness landscape in Fig. 3 does indeed represent the fitness landscape experienced by an evolving metapopulation of a single species.

As shown in Fig. S3, $\overline{\log_{10} a}$ increases linearly with p for low p. An occupancy of $p = 0.3$, for example, leads to an approximate 3-fold increase in geometric mean base attack rates. Hence, cheating makes consumers somewhat less prudent, but does not fundamentally undermine the evolution of prudent predation.

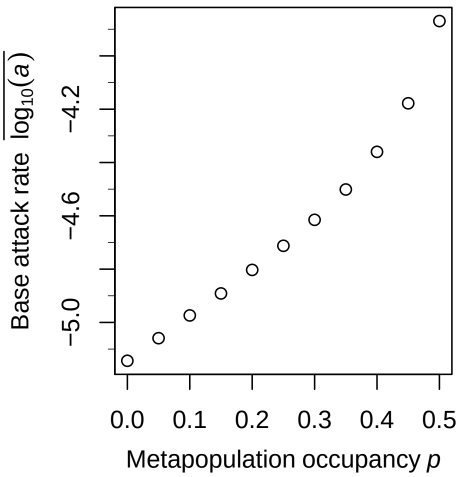

Figure S3: **The impact of cheaters on evolutionary stable base attack rate** a**.** Simulation results from the metapopulation model described in Box S1. The higher the occupancy p of patches by the metapopulation, the larger the probability that occupied patches are overtaken by invading cheaters with higher base attack rates. This effect increases steady state base attack rates but does not prevent a steady state from being reached.

Appendix S7 Steepness and basic reproduction number

We derive the relation between basic reproduction number and the steepness of stock recruitment relations given in Eq. (12).

Consider first the following caricature model of a fish stock with standing stock biomass SSB that feeds on a single resource:

$$\frac{dB^{\mathrm{R}}}{dt} = \left[s \left(1 - \frac{B^{\mathrm{R}}}{K} \right) - a\,\mathrm{SSB} \right] B^{\mathrm{R}}, \tag{S35a}$$

$$\frac{d\,\mathrm{SSB}}{dt} = \epsilon a B^{\mathrm{R}} \mathrm{SSB} - \rho\,\mathrm{SSB} - F\,\mathrm{SSB}. \tag{S35b}$$

The parameter F denotes the fishing mortality rate, otherwise model structure and parameterization are as in Eq. (1). If one assumes, for simplicity, that (i) all mature individuals have the same body mass m, (ii) recruits are produced instantaneously, and (iii) the parameter ρ is dominated by natural mortality rather than respiration, then recruitment is given by the first term on the right-hand side of Eq. (S35b):

$$m\,\mathrm{Rec} = \epsilon a B^{\mathrm{R}} \mathrm{SSB} = \epsilon a K\,\mathrm{SSB} - \frac{\epsilon a^2 K}{s} \mathrm{SSB}^2. \tag{S36}$$

In the second step we eliminated B^{R} by solving Eq. (S35a) with $dB^{\mathrm{R}}/dt = 0$ for $B^{\mathrm{R}} > 0$. Stock-recruitment relations of this quadratic form are frequently used in fisheries science and named after Schaefer (1954). Unfished ($F = 0$) equilibrium SSB evaluates to

$$\mathrm{SSB}_0 = s \frac{\epsilon a K - \rho}{\epsilon a^2 K}. \tag{S37}$$

From Eqs. (11), (S36) and (S37) one obtains the steepness

$$h = \frac{1}{25} \left(1 + \frac{4 \epsilon a K}{\rho} \right). \tag{S38}$$

The basic reproduction number R is defined as recruitment per mature individual (of which there are SSB/m) in units of ρ, in the limit $\mathrm{SSB} \to 0$, which evaluates to

$$R = \lim_{\mathrm{SSB} \to 0} \frac{m\mathrm{Rec}}{\mathrm{SSB}\,\rho} = \frac{\epsilon a K}{\rho}. \tag{S39}$$

Hence Eq. (S38) implies Eq. (12).

We now verify that Eq. (12) remains valid if one generalises Eq. (S35) to a situation with multiple resources. We assume that the fish stock is initially fully established at SSB_0, such that resources that would not withstand its consumption have been extirpated. By Eq. (S6), the biomass of each resource is then a linear function of consumer biomass, here SSB. With the linear functional response of Lotka-Volterra models, this implies

$$m\,\mathrm{Rec} = (c_1 - c_2\,\mathrm{SSB})\,\mathrm{SSB} \tag{S40}$$

with some positive constants c_1 and c_2. As above, we can evaluate

$$R = \lim_{\mathrm{SSB} \to 0} \frac{m\mathrm{Rec}}{\mathrm{SSB}\,\rho} = \frac{c_1}{\rho}, \tag{S41}$$

yielding $c_1 = \rho R$. Furthermore, recruitment balances mortality for an unfish stock with $\mathrm{SSB} = \mathrm{SSB}_0$. So $m\,\mathrm{Rec}(\mathrm{SSB}_0) = \rho\,\mathrm{SSB}_0$, which implies

$$c_2 = \rho \frac{R - 1}{\mathrm{SSB}_0}. \tag{S42}$$

With these values for c_1, c_2, plugging Eq. (S40) into the definition of steepness, Eq. (11), yields again Eq. (12).

Supporting figures

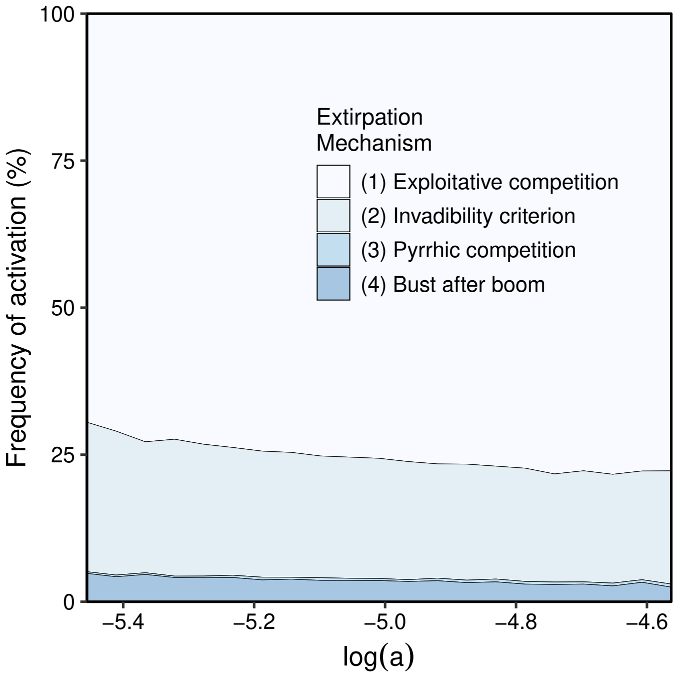

Figure S4: **Activation frequency of consumer extirpation mechanisms.** The classification relates to different steps in the deconstructed model formulation (Box 1). *Exploitative competition* refers to Step 5; *Pyrrhic competition* to failure to meet the invasibility condition by a consumer losing its main resource, or by the competitor causing this, in Steps 6, 7; *Bust after boom* refers to Step 3d; and *Invasibility criterion* to failure to satisfy Eq. (13) at any other point in the algorithm. Extirpations through Pyrrhic competition are very rare, and those through bust after boom contribute just a few percent of cases.

References

Bersier, L.F. & Kehrli, P. (2008). The signature of phylogenetic constraints on food-web structure. *Ecol. Complex.*, 5, 132–139.

Case, T.J. & Casten, R.G. (1979). Global stability and multiple domains of attraction in ecological systems. *Am. Nat.*, 5, 705–714.

Chesson, P. (1990). MacArthur's consumer-resource model. *Theor. Popul. Biol.*, 37, 26–38.

Eklöf, A. & Stouffer, D.B. (2016). The phylogenetic component of food web structure and intervality. *Theor. Ecol.*, 9, 107–115.

Goodnight, C., Rauch, E., Sayama, H., de Aguiar, M.A.M., Baranger, M. & Bar-Yam, Y. (2008). Evolution in spatial predator-prey models and the "prudent predator": The inadequacy of steady-state organism fitness and the concept of individual and group selection. *Complexity*, 13, 23–44.

Holt, R.D. (1977). Predation, apparent competition, and the structure of prey communities. *Theor. Popul. Biol.*, 12, 197–229.

Levins, R. (1969). Some Demographic and Genetic Consequences of Environmental Heterogeneity for Biological Control. *Bull. Entomol. Soc. Am.*, 15, 237–240.

Lotka, A.J. (1920). Undamped oscillations derived from the law of mass action. *J. Am. Chem. Soc.*, 42, 1595–1599.

MacArthur, R. (1970). Species packing and competitive equilibrium for many species. *Theor. Popul. Biol.*, 1, 1–11.

MacArthur, R.H. (1972). *Geographical Ecology.* Harper and Row, New York.

Nagelkerke, L.A.J. & Rossberg, A.G. (2014). Trophic niche-space imaging, using resource and consumer traits. *Theor. Ecol.*, 7, 423–434.

O'Sullivan, J.D., Knell, R.J. & Rossberg, A.G. (2019). Metacommunity-scale biodiversity regulation and the self-organised emergence of macroecological patterns. *Ecol. Lett.*, 22, 1428–1438.

Page, K.M. & Nowak, M.A. (2002). Unifying evolutionary dynamics. *J. Theor. Biol.*, 219, 93–98.

Pomiankowski, A., Iwasa, Y. & Nee, S. (1991). The evolution of costly mate preferences I. Fisher and biased mutation. *Evolution*, 45, 1422–1430.

Price, G.R. (1972). Extension of covariance selection mathematics. *Ann. Hum. Genet.*, 35, 485–490.

Rohr, R.P., Scherer, H., Kehrli, P., Mazza, C. & Bersier, L.F. (2010). Modeling food webs: Exploring unexplained structure using latent traits. *Am. Nat.*, 176, 170–177.

Rossberg, A.G. (2007). Some first principles of complex systems theory. *RIMS Kôkyûroku*, 1551, 129–136.

Rossberg, A.G. (2013). *Food Webs and Biodiversity: Foundations, Models, Data.* Wiley.

Rossberg, A.G., Brännström, A. & Dieckmann, U. (2010). How trophic interaction strength depends on traits — A conceptual framework for representing multidimensional trophic niche spaces. *Theor. Ecol.*, 3, 13–24.

Rossberg, A.G., Farnsworth, K.D., Satoh, K. & Pinnegar, J.K. (2011). Universal power-law diet partitioning by marine fish and squid with surprising stability-diversity implications. *Proceeding R. Soc. B*, 278, 1617–1625.

Rossberg, A.G., Matsuda, H., Amemiya, T. & Itoh, K. (2006). Food webs: Experts consuming families of experts. *J. Theor. Biol.*, 241, 552–563.

Schaefer, M.B. (1954). Fisheries dynamics and the concept of maximum equilibrium catch. In: *Proceedings of the Gulf and Caribbean Fisheries Institute - 6th Annual Session.* pp. 53–64.
